# “Golden cicada-escape” style colon-targeted pellets for ulcerative colitis by balancing oxidative stress and repairing colonic barrier

**DOI:** 10.1016/j.mtbio.2025.102608

**Published:** 2025-11-25

**Authors:** Zihan Liu, Shan Chen, Jinghan Yu, Zhiyang Wen, Yue Gao, Yanfang Yang, Hongliang Wang, Qingce Zang, Jun Ye, Yuling Liu

**Affiliations:** a State Key Laboratory of Bioactive Substance and Function of Natural Medicines, Institute of Materia Medica, Chinese Academy of Medical Sciences & Peking Union Medical College, Beijing, 100050, PR China; b Beijing Key Laboratory of Drug Delivery Technology and Novel Formulation, Institute of Materia Medica, Chinese Academy of Medical Sciences & Peking Union Medical College, Beijing, 100050, PR China; c Beijing Wehand-Bio Pharmaceutical Co., Ltd., Beijing, 100260, PR China

**Keywords:** Ulcerative colitis, Colon-targeted pellets, pH-sensitive, Mucosal adhesion, Golden cicada-escape, Oxidative stress

## Abstract

Ulcerative colitis (UC) is a chronic, recurrent inflammatory disease of the colon with a complex and unknown etiology. Oxidative stress and impaired colonic barrier function are key factors in the pathogenesis of UC. *Ramulus Mori* (Sangzhi) Alkaloids (SZ-A), a group of natural plant-derived compounds, exhibit multiple pharmacological activities, including modulating anti-inflammatory immune responses, attenuating oxidative stress, and restoring intestinal barrier integrity. These properties highlight their great potential in the treatment of UC. However, SZ-A is prone to intestinal flatulence after oral administration due to its interaction with α-glucosidase in the small intestine, and high doses may pose a risk of weight loss, which is detrimental to patients with UC. To address these challenges, this study developed a pH-sensitive, colon-targeted pellet with colonic mucosal adhesion for the oral delivery of SZ-A, representing the inaugural successful application of this approach in the treatment of UC. The pH sensitivity and mucosal adhesion properties of the system were validated through both *in vivo* and *in vitro* experiments. Under colonic pH conditions, the outer shells of the pellets dissolved and were completely dislodged, thereby exposing the pellet cores and achieving the “golden cicada-escape” effect. The pellet cores rapidly adhered to and aggregated at the colonic lesion site due to the action of chitosan, followed by a slow release of SZ-A. The colon-targeted adhesion pellets effectively mitigated the progression of UC in rats through multiple mechanisms, including anti-inflammatory effects, reduction of oxidative stress, repair of the intestinal barrier, and optimization of intestinal flora composition. This system offers a precise, efficient, and translationally promising delivery strategy for the oral treatment of UC and is anticipated to be an ideal clinical candidate.

## Graphical abstract

**Figure:**
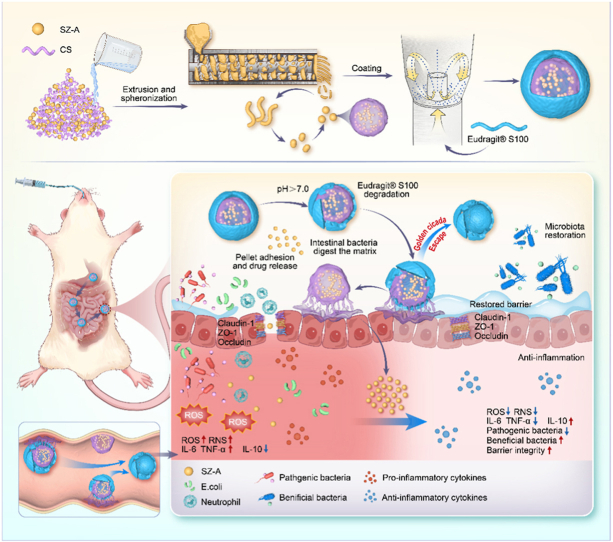

## 1 Introduction

Ulcerative colitis (UC) is a lifelong, recurrent autoimmune disorder characterized by clinical symptoms including hematochezia and weight loss [1]. Intestinal epithelial cells are crucial in maintaining intestinal homeostasis by providing chemical and physical barriers that separate intestinal microorganisms from the mucosal immune system, regulating paracellular selective permeability, and preserving epithelial barrier integrity [2]. The pathogenesis of UC is related to multiple factors such as leakage of the intestinal epithelial barrier, dysregulation of the mucosal immune response, oxidative stress induced by microbial antigens, and alterations in the expression and function of connexins within the inflammatory microenvironment [3]. Recent advances in nanotherapeutic strategies have shown promise for UC treatment. For instance, mulberry biomass-derived nanomedicines demonstrate enhanced inflamed mucosa accumulation and intestinal microenvironment modulation [4], while thiol-disulfide exchange systems enable coordinated drug release for synergistic regulation of colitis [5]. Despite these innovations, current small molecules and biologics used for UC treatment still demonstrate limited efficacy and serious adverse effects, failing to adequately address clinical needs.

*Ramulus Mori* (Sangzhi) Alkaloids (SZ-A) is a group of polyhydroxy-alkaloid effective components extracted and isolated from the mulberry branch and has received approval for the treatment of type 2 diabetes mellitus (T2DM) in China (Approval No. Z20200002) [6]. Beyond its hypoglycemic properties, SZ-A has demonstrated multiple pharmacological effects, such as mitigating intestinal inflammation and oxidative stress, modulating the composition of intestinal flora, and repairing the intestinal barrier [7]. These findings suggest its significant potential and favorable safety profile for the treatment of UC. In models of UC induced by Dextran Sulfate Sodium (DSS), SZ-A markedly ameliorated symptoms such as body weight loss, diarrhea, bloody stools, and colonic atrophy, while also reducing the Disease Activity Index (DAI) in rats. A thermo-sensitive SZ-A hydrogel designed for injectable and adherent rectal delivery has been developed to effectively mitigate 2,4,6-Trinitrobenzenesulfonic acid (TNBS)-induced UC in rats [8]. However, rectal delivery poses challenges in precisely targeting the proximal colon and is often difficult for patients to self-administer, leading to low compliance. Conversely, oral administration is the most prevalent mode of drug delivery due to its non-invasive nature and superior safety and clinical benefits. Nonetheless, oral administration of SZ-A for UC presents challenges, such as intestinal bloating due to interactions with α-glucosidase and the potential risk of weight loss at high doses a), d). Consequently, there is an urgent need to develop an oral, colon-targeted delivery system for SZ-A to prevent its release in the stomach and small intestine, thereby enhancing efficacy, reducing dosage, and minimizing side effects.

Among the various intelligent colon-targeted delivery systems designed based on the colon's unique physiological and pathological characteristics, the pH-dependent colon-targeted drug release system offers advantages such as simplicity, mature technology, stability, and reliability [9]. Polymers that exhibit water insolubility and impermeability at low pH levels are commonly employed as coating materials, including Eudragit (S100, L30D), cellulose, and its derivatives. Additionally, natural polysaccharides are extensively utilized due to their abundant availability, biocompatibility, and cost-effectiveness. These materials enhance the efficiency of oral drug delivery to the colon through mechanisms such as pH responsiveness, gastric resistance, and degradability by intestinal microbes. Certain polysaccharides undergo fermentation by the intestinal microbiota, functioning as prebiotics and bolstering intestinal mucosal protection by modulating inflammatory mediators, microbial flora, and the immune system, thereby presenting potential therapeutic benefits for UC [10]. Currently, polysaccharides such as chitosan, inulin, alginate, and hyaluronic acid are extensively investigated in the context of oral colon-targeted delivery systems [11]. However, for water-soluble, multi-component, small-molecule drugs, these systems present several challenges, including low encapsulation efficiency, susceptibility to leakage, instability, and difficulties in scaling up for industrial production.

**Scheme 1:**
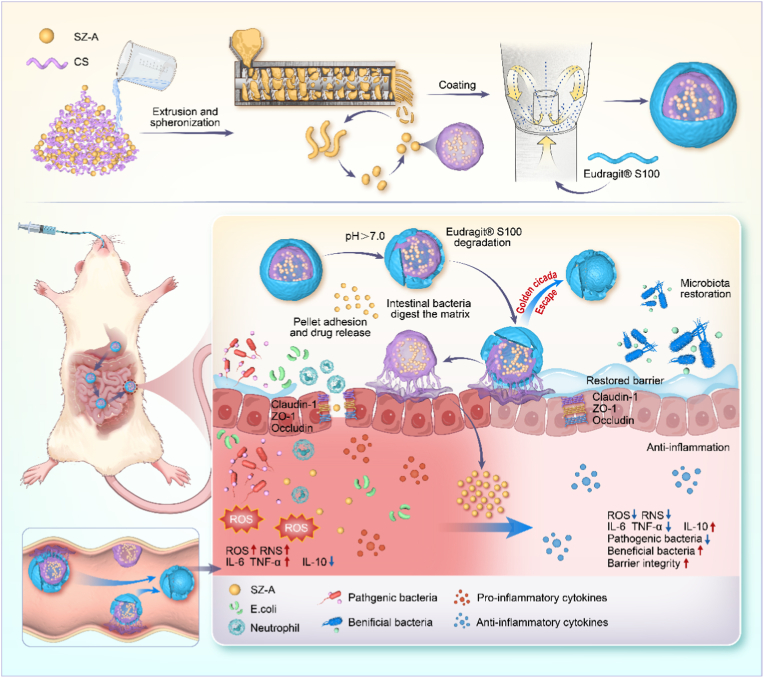
“Golden cicada-escape” style colon-targeted pellets for ulcerative colitis by balancing oxidative stress and repairing the colonic barrier.

In this study, we employed the concept of a multi-unit pellet delivery system to develop colon-targeted adhesion oral pellets containing SZ-A in a manner reminiscent of the “Golden cicada-escape” strategy. Specifically, the pellet cores, which contained SZ-A and functional polysaccharides (chitosan or sodium alginate), were fabricated using an extrusion-spheronization technique. Subsequently, a coating shell of controlled thickness was applied to the exterior of the pellet cores via bottom spraying in a fluidized bed, utilizing Eudragit® S100 as the primary component. Upon oral administration, the coating shell is designed to withstand the harsh conditions of the stomach and small intestine. Once the pellets reach the colon, the shell dissolves under the pH conditions present, allowing for the rapid and complete exposure of the pellet cores to the colonic environment, thereby achieving the intended “Golden cicada-escape” mechanism. The polysaccharides facilitated the adhesion of the pellet cores to the colonic mucosal tissue, thereby extending their local retention time and sustaining their efficacy for targeted colon-specific oral delivery. The study demonstrated the pivotal role of SZ-A pellets in ameliorating intestinal dysfunction through comprehensive modulation of the intestinal microbiota, anti-inflammatory and antioxidant activities, and enhancement of the colonic intestinal mucosal barrier (see Scheme 1). This research provides valuable insights into a potential strategy for UC treatment and offers scientific support for developing a water-soluble, multi-component, small-molecule drug delivery system, as well as the clinical application of SZ-A.

## 2 Materials and methods

### 2.1 Materials

The SZ-A extract, microcrystalline cellulose (MCC) PH101, calcium phosphate (CaHPO_4_), and mannitol (Man) were generously provided by Beijing Wehand-Bio Pharmaceutical Co., Ltd. (Beijing, China). Chitosan (CS, degree of deacetylation>70 %) was procured from Hunan Xinlvfang Pharmaceutical Co., Ltd. (Changsha, China). Eudragit® S100 (methacrylic acid and methyl methacrylate 1:2) was sourced from Evonik (Shanghai, China). Sodium alginate (SA, M/G = 1:1, viscosity 200 ± 20 mPa S) and new indocyanine green IR-820 were obtained from Macklin (Shanghai, China). The 2,4,6-Trinitrobenzenesulfonic acid solution (TNBS, 5 % in H_2_O) was purchased from Shanghai Acmec Biochemical Technology Co., Ltd. (Shanghai, China). Dextran sodium sulfate (DSS, molecular weight 36–50 kDa) was acquired from Yeasen (Shanghai, China). 5-Aminosalicylic acid (5-ASA) with a purity of 95 % was procured from Sigma-Aldrich (MO, USA). A rat myeloperoxidase (MPO) enzyme-linked immunosorbent assay (ELISA) kit was obtained from Cusabio (Wuhan, China). The superoxide dismutase (SOD) assay was sourced from Nanjing Jiancheng Bioengineering Institute (Nanjing, China). Monoclonal antibodies against Claudin-1 and ZO-1 were purchased from Abcam (Cambridge, U.K.), and a polyclonal antibody against Occludin was obtained from Proteintech (Wuhan, China). All other reagents and solvents used were of analytical grade.

### 2.2 Preparation of SZ-A-loaded pellet cores

Pellet cores containing SZ-A were formulated via extrusion-spheronization. The formulation was systematically optimized through single-factor experiments evaluating key variables such as the microcrystalline cellulose-to-mannitol ratio, SZ-A extract content, wetting agent type, polysaccharide type, and polysaccharide concentration. These were assessed based on critical quality attributes including sphericity, particle size distribution (yield), *in vitro* adhesiveness, and drug release profile. This optimization process yielded the final composition containing SZ-A extract (30 %), microcrystalline cellulose (40 %), chitosan (20 %), and calcium phosphate (10 %). The powdered components were uniformly before being moistened with 50 % ethanol to form a mass with suitable plasticity.

Extrusion and spheronization were performed using an experimental multifunctional coating machine(Mini-250, Shenzhen Xinyite Technology Co., Ltd., China). Process parameters including extrusion speed, spheronization speed, and spheronization time were systematically optimized via single-factor experiments targeting pellet yield, sphericity, and size distribution. The mass was extruded through a 0.8 mm sieve to produce strips of wet mass at a rotational speed of 50 r/min and 4 °C, then spheronized at 1000 r/min for 3–5 min. The resulting pellets were dried at 45 °C for 2 h.

Pellet cores with diameters of 0.6–0.9 mm, designated as CS@SZ-A@pellet cores, were collected. The yield was determined as the mass percentage of cores within this size range. Samples substituted for CS with SA or Man were also prepared as controls and designated as SA@SZ-A@pellet cores and Man@SZ-A@pellet cores, respectively. All samples were sealed and stored for subsequent experimentation.

### 2.3 Preparation of CS@SZ-A@coated pellets

The coating formulation and process parameters were optimized using a single-factor experimental approach. Critical variables in the coating formulation, including the content of pH-responsive polymer, plasticizer, and anti-tacking agent, as well as key process parameters such as coating solution preparation method, batch size, product temperature, peristaltic pump speed (spray rate), bottom-spray pressure, and atomization pressure, were systematically evaluated. The optimization was based on the quality of the resulting film coat, particularly surface smoothness and the absence of pellet agglomeration during the coating process. This method successfully identified the optimal coating formulation and processing conditions. For the outer coating, a Eudragit® S100 dispersion (7.5 %, w/v) was applied to CS@SZ-A@pellet cores using a bottom-sprayed fluidized bed apparatus (Glatt, Germany) until a 100 % total weight gain (TWG) was achieved, producing the final CS@SZ-A@coated pellets. The TWG was calculated using Equation (1). For comparative purposes, additional products were prepared with various pellet core types and TWGs of 20 %, 40 %, 60 % and 80 %.

The coating process employed the following parameters: peristaltic pump speed of 1.5 r/min, inlet temperature of 25 °C, bottom-spray pressure of 1.0 bar, and atomization pressure of 0.8 bar.

(1) $$T\text{WG}\left(\%\right)=\frac{\text{weight}\;\text{after}\;\text{coating}-\text{weight}\;\text{before}\;\text{coating}}{\text{weight}\;\text{before}\;\text{coating}}*100\%$$

### 2.4 Size and morphology characterization

The scanning electron microscopy (SEM, FEI Apreo, Thermo, USA) was employed to characterize the shape, surface morphology, and cross-sectional morphology of the samples. An image analysis system was subsequently utilized to ascertain the size and shape of the particles. A total of 100 pellets were randomly selected, and image capture was conducted using ImageJ software to facilitate the analysis of various parameters, including perimeter and area. The form factor (F) was calculated according to Equation (2), with F reaching a minimum value of 1 when the area was circular.

(2) $$F=B^{ˆ}\left(2\right)\;/\left(4\pi A\right)$$

Here, B represents the perimeter and A represents the area.

### 2.5 Adhesion of SZ-A-loaded pellet cores *in vitro*

The tissue retention method was employed to assess the *in vitro* adhesion of the samples. To simulate *in vivo* conditions, fresh rat colon tissue was excised and dissected, and 50 mg of the sample was uniformly distributed on the tissue. This setup was then placed in a sealed container with a saturated KNO_3_ environment (92 % relative humidity, room temperature) for 20 min to ensure adequate hydration and interaction with glycoproteins (colonic mucosa) while preventing mucus desiccation. At the commencement of the assay, the intestinal tissue and samples were inclined at 45° and rinsed with an artificial colonic solution at a flow rate of 22 mL/min for 5 min. The percentage of pellets remaining on the mucosa was calculated according to Equation (3). The higher the number of remaining pellets, the better the *in vitro* adhesion.

(3) Pellet residue (%) = (number of pellets after rinsing / total number of pellets) ∗ 100 %

### 2.6 *In vitro* release analysis

Buffer solutions with different pH values were employed at distinct time intervals to simulate the drug undergoing different pH and transport times within the gastrointestinal tract. The samples were initially immersed in 250 mL of artificial gastric fluid (pH 2.0, containing enzymes) and agitated at 37 °C for 2 h. Subsequently, they were transferred to 250 mL of artificial small intestine fluid (pH 6.8) for 3 h, followed by a transfer to 250 mL of artificial colon fluid (pH 7.8) for 7 h. At predetermined intervals, 5 mL of the release medium was extracted, and an equivalent volume of fresh medium was introduced. The cumulative drug release was quantified using Equations (4), (5) to evaluate the extent of drug release from various formulations.

(4) $$\text{Cumulative}\;\text{release}\left(\%\right)=\left(Q_{n}/Q_{0}\right)*100\%$$

(5) $$Q_{n}=c_{n}V_{n}+\sum_{i=0}^{n-1}c_{i}V_{i}$$

Where Q_0_ represents the total drug content in the pellets, c_n_ denotes the measured concentration of the drug in the nth sampled test solution, V_n_ is the total volume of the release medium, c_i_ signifies the measured concentration of the drug in the ith test solution, and V_i_ is the volume of each sampling.

Mathematical models were used to validate the drug release mechanism of the pellets. The *in vitro* drug release data were fitted to various models, including the zero-order release model, first-order release model, Higuchi model, Ritger-Peppas model, Logistic model, Hill model, Boltzmann model, and Gompertz model, using Origin 2024b software. The resulting fitted equations and coefficients of determination (R^2^) were obtained.

### 2.7 CT-based *in vitro* release

Further, off-line computerized tomography (CT) testing equipment (Beijing Gongyuansanqian Technology Co., LTD.) was utilized to investigate the internal microstructure changes and drug release mechanism during pellet dissolution. The scanning parameters were as follows: voltage of 50 kV, power of 22 W, scanning angle of 360°, 1800 scanned images, and a scanning duration of 30 min.

### 2.8 Distribution and form of drugs

The distribution of components within the pellets was examined using a non-contact sub-micron resolved infrared Raman simultaneous measurement system, mIRage™ (Quantum Design, USA), based on Optical Photothermal Infrared (O-PTIR) technology.

The form of SZ-A present in the pellets was analyzed via powder X-ray diffraction (XRD, Bruker, Germany), with samples exposed to Cu-Kα radiation and analyzed over a diffraction angle range of 3°–90° at a scan rate of 5°/min.

### 2.9 Animals

All experiments were approved by the Laboratories’ Institutional Animal Care and Use Committee of the Chinese Academy of Medical Sciences, and were carried out in accordance with the requirements of regulations such as the “Regulations on Experimental Animals of Beijing Municipality” (animal experiment ethical approval number: 00001846, 00002854; facility use certificate for experimental animals: 0004254, 0004256).

Male Sprague-Dawley (SD) rats aged 6–8 weeks (200–250 g) were procured from Beijing Huafukang Biotechnology Co., Ltd. The rats were housed in a specific pathogen-free (SPF) environment and subjected to a 12 h light/dark cycle at 22–25 °C. Prior to experimentation, the rats underwent a one-week acclimatization period, after which they were randomly assigned to various experimental groups.

### 2.10 *In vivo* targeting and retention of CS@SZ-A@coated pellets in the colon

To assess the colonic targeting and retention of CS@SZ-A@coated pellets *in vivo*, we utilized IR-820 fluorescent dye to prepare fluorescently labeled coated pellets. Healthy rats were divided into 5 groups, each comprising 3 rats. These groups were administered different formulations: a free IR-820 solution (Free IR-820), IR-820-loaded pellet cores (0 %), and IR-820-loaded coated pellets with weight gains of 20 %, 60 %, and 100 % (denoted as 20 %, 60 %, and 100 %, respectively). The administered fluorescence dose of IR-820 was 0.75 mg/kg. An *in vivo* imaging system for small animals (IVIS Spectrum CT, Caliper-Perkin Elmer, USA) with an excitation wavelength of 745 nm and an emission wavelength of 840 nm was employed to investigate the *in vivo* distribution of the drug over a 24 h period. Following the final imaging session, the gastrointestinal tract was excised to examine the retention and distribution of fluorescence *in vivo*.

### 2.11 Tissue distribution based on AFADESI-MSI

To analyze the distribution of a specific component of SZ-A within tissues, 12 rats were allocated into 4 groups. The normal control group (referred to as Normal) received no treatment and other rats were administered a gavage of 50 mg/kg SZ-A in the forms of Free SZ-A (referred to as Free SZ-A), CS@SZ-A@pellet cores (referred to as 0 %), and CS@SZ-A@coated pellets (referred to as 100 %). The animals were subsequently sacrificed after 6 h. After liquid nitrogen fixation, the pellets were stored at −80 °C. Subsequently, the entire animal underwent cryosectioning parallel to the abdomen, and all tissue sections were dried in a vacuum desiccator at −20 °C for 2 h and at room temperature for an additional 2 h prior to analysis using air flow-assisted desorption electrospray ionization-mass spectrometry imaging (AFADESI-MSI).

### 2.12 The therapeutic effect of CS@SZ-A@coated pellets on TNBS-induced colitis rats

The rats were randomly assigned to 6 groups, each comprising 6 individuals: (1) a blank control group (Normal) that received no treatment; (2) a model control group (TNBS) administered with the corresponding volume of 1 % sodium carboxymethyl cellulose (CMC-Na) solution via gavage; (3) a positive drug treatment group receiving 5-ASA suspension (5-ASA, 100 mg/kg); (4) a treatment group receiving SZ-A solution (Free SZ-A, 34.62 mg/kg, as SZ-A); (5) a low-dose CS@SZ-A@coated pellets treatment group (L-SZ-A, 17.31 mg/kg, as SZ-A, administered in 1–1.5 mL of 1 % CMC-Na solution); and (6) a medium-dose CS@SZ-A@coated pellets treatment group (M-SZ-A, 34.62 mg/kg, as SZ-A, administered in 1–1.5 mL of 1 % CMC-Na solution). The UC model in rats was induced using TNBS in all groups except the Normal group. Following anesthesia, a 5 % aqueous solution of TNBS mixed with anhydrous ethanol at a ratio of 2.5:1 (v/v) was slowly injected into the rats' anus at a dose of 60 mg/kg. The day of model induction was designated as day 0. Commencing on day 1, the drug was administered via gavage once daily for 7 consecutive days, during which all rats had unrestricted access to food and water.

Rats were euthanized 24 h after the last administration, and samples of whole blood, serum, colon, heart, liver, spleen, lung, kidney, thymus, and colon contents were collected for subsequent analysis.

### 2.13 The therapeutic effect of CS@SZ-A@coated pellets on DSS-induced colitis rats

The efficacy of CS@SZ-A@coated pellets was further validated using a DSS-induced UC model in rats. The rats were randomly allocated into 5 groups, each comprising 6 rats: (1) a blank control group (Normal) that received no treatment; (2) a model control group (DSS); (3) a positive control group treated with a 5-ASA suspension (5-ASA, 100 mg/kg); (4) a group treated with an SZ-A solution (Free SZ-A, 17.31 mg/kg, based on SZ-A content); and (5) a low-dose CS@SZ-A@coated pellet treatment group (L-SZ-A, 17.31 mg/kg, as SZ-A). The Normal group was provided with regular drinking water, while the other groups received 5 % DSS in their drinking water ad libitum for 5 days. Drug administration commenced on day 1 and continued once daily for 10 days. Twenty-four hours following the final administration, all rats were euthanized, and blood and colon samples were collected for subsequent analyses.

The rats were weighed at a consistent time each morning and monitored for changes in coat condition, activity levels, fecal consistency, and the presence of bleeding. DAI scores were calculated by integrating the percentage of weight loss, fecal consistency, and fecal bleeding (as detailed in Table S1) [12]. A higher DAI score indicates a greater severity of the disease.

Following euthanasia, the animals were quickly dissected from the cecum to the colon, with meticulous removal of surrounding adipose and connective tissues. The colons of each group of rats were then placed on a clean background plate, neatly arranged for length measurement, and photographed.

The concentrations of IL-6, TNF-α, IL-1α, and MPO activity in colonic tissues, along with serum levels of IL-6, TNF-α, and IL-2, were quantified using the respective ELISA kits (Sino Biological, Beijing, China). The IL-10 and TGF-β1 were measured by a nanoparticle protein detection luminescence system (Biosolution, Beijing, China).

Additionally, levels of hydrogen peroxide (H_2_O_2_), nitric oxide (NO), malondialdehyde (MDA), SOD, catalase (CAT), glutathione (GSH), and total antioxidant capacity (T-AOC) in colon tissues were assessed using a commercial assay kit (Beijing LABLEAD Inc.) as per the manufacturer's protocol.

### 2.14 Histological analysis (HE, AB/PAS), IHC, IF, and TUNEL assay

The colonic tissues were fixed in 4 % paraformaldehyde, embedded in paraffin, and sectioned into 4 μm thick slices. Hematoxylin and eosin (H&E) staining (Solarbio, Beijing, China) was employed to examine the histological morphology of the colon under a bright-field microscope, facilitating the analysis of morphological lesions. Alcian blue/periodic acid-Schiff (AB/PAS) staining was conducted to evaluate the number of goblet cells and alterations in the mucus layer. Terminal deoxynucleotidyl transferase-mediated dUTP nick end labeling (TUNEL) staining was conducted to assess apoptosis in hindgut cells, following established protocols. Immunohistochemistry (IHC) and immunofluorescence (IF) techniques were employed to evaluate the protein expression levels of Claudin-1, ZO-1, and Occludin in colonic tissues, thereby assessing intestinal barrier function.

### 2.15 16S rDNA sequencing of intestinal microbiota

Fresh fecal samples were collected from rats, rapidly frozen in liquid nitrogen, and subsequently stored at −80 °C. Novogene Co., Ltd. was engaged to perform 16S rDNA amplicon sequencing for the identification and analysis of the intestinal bacterial composition in each sample.

### 2.16 *In vivo* safety evaluation

The preliminary safety assessment of CS@SZ-A@coated pellets involved performing routine blood analyses, calculating major organ indices and performing serum biochemical tests. After the treatment period, blood samples were obtained from the abdominal main vein of the rats and collected in EDTA-containing tubes. Routine blood analyses were then immediately conducted using a fully automated blood cell analyzer (Myeri, BC5000 Vet, China). The hearts, liver, spleen, lungs, kidneys, and thymus of rats were collected, weighed, photographed, and documented. The organ weight-to-body weight ratio was calculated to determine the corresponding organ index. Serum biochemical parameters including aspartate aminotransferase (AST), alanine aminotransferase (ALT), urea, creatinine (Cr), creatine kinase (CK), and lactate dehydrogenase (LDH) were measured using standard automated biochemical analyzers (HITACHI, 7100, Japan).

### 2.17 Statistical analysis

Data are presented as mean ± standard error of the mean (S.E.M.). Statistical analyses were conducted using GraphPad Prism Software Version 9.0 (San Diego, CA, USA) or Origin 2024b. Differences between groups were evaluated using one-way ANOVA, with statistical significance set at p < 0.05. Significance levels were denoted as follows: ∗p < 0.05, ∗∗p < 0.01, ∗∗∗p < 0.00.

## 3 Results and discussion

### 3.1 Preparation and characterization of CS@SZ-A@coated pellets

**Fig. 1:**
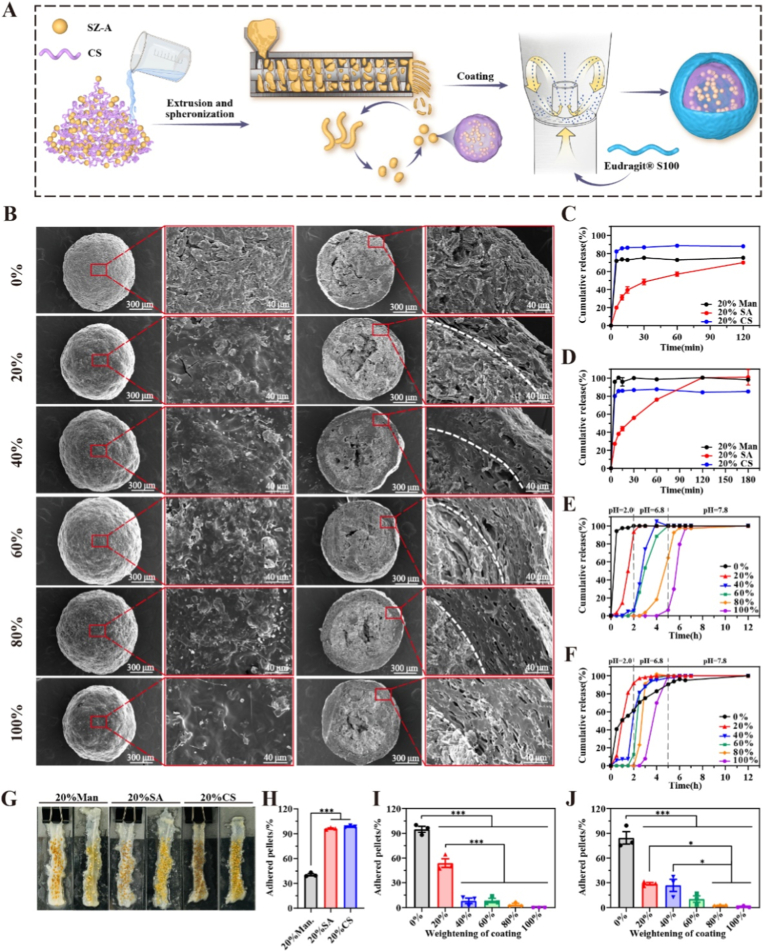
Preparation process and characterization of CS@SZ-A@coated pellets. (A) Colon-targeted pellets prepared by extrusion-spheronization and bottom-sprayed fluidized bed coating. (B) Representative SEM images of CS@SZ-A@coated pellets with different TWG. Dissolution experiments of CS@SZ-A@pellet cores in (C) artificial gastric fluid and (D) artificial colonic fluid (n = 3). (E) *In vitro* release profiles of coated pellets with different TWG containing SA@SZ-A@pellet cores and (F) CS@SZ-A@pellet cores. At the same TWG, the coated pellets containing CS@SZ-A@pellet cores were more resistant to gastric and small intestinal fluids. *In vitro* adhesion of 3 different pellet cores, (G) comparative representative images before and after rinsing, and (H) percentage of remaining pellets (n = 3). *In vitro* adhesion of coated pellets with different TWG (I) containing SA@SZ-A@pellet cores and (H) CS@SZ-A@pellet cores (n = 3). Data were expressed as mean ± S.E.M. and were significant by one-way ANOVA: ∗p < 0.05, ∗∗p < 0.01, ∗∗∗p < 0.001.

SZ-A, the active ingredient in the extract, constitutes more than 50 % of the composition [7a]. Sieve analysis (Fig. S1) revealed that CS@SZ-A@pellet cores exhibited a narrow particle size distribution, with 87.49 ± 6.5 % of particles within the 0.6–0.9 mm range (n = 3), and demonstrated a high yield. Particles smaller than 0.6 mm accounted for 10.40 ± 5.55 % (n = 3), while agglomerates larger than 0.9 mm constituted 2.11 ± 1.8 % (n = 3). Cores within the 0.6–0.9 mm range were selected for subsequent coating to ensure a more uniform drug release rate. In the fluidized bed, approximately 10 g of pellet cores exhibit a vortex-like motion due to the upward inlet airflow, which subsequently propels them into the spray chamber. Here, a coating solution is applied to the pellet surfaces, initially forming a thin film that eventually envelops the entire surface, resulting in colon-targeted CS@SZ-A@coated pellets (Fig. 1A).

The incorporation of 20 % CS or 20 % SA did not compromise the sphericity of the pellet cores, which predominantly retained a complete spherical shape (Fig. S2). SEM was employed to examine the morphology of the pellet surface and cross-section. Fig. 1B presents the SEM image of an uncoated pellet core, which is spherical with a rough surface devoid of cracks. The cross-section reveals a porous internal structure. Post-coating, the pellet surfaces became smoother, and the cross-section displayed a distinct bright line separating the coating layer from the pellet core. The coating layer exhibited increased density, and its thickness was observed to augment the increase in the TWG of the coating. The parameter of the shape factor serves as an indirect measure of roundness. The shape factors for Man@SZ-A@pellet cores, SA@SZ-A@pellet cores, and CS@SZ-A@pellet cores were approximately 1, with values of 1.09 ± 0.06, 1.08 ± 0.05, and 1.10 ± 0.06, respectively. These values suggest that the shapes of the pellet cores closely approximate a circle, indicating good roundness. Consequently, the pellet cores exhibited a uniform size distribution and adequate roundness, both of which are critical for effective drug release control.

### 3.2 Adhesion of SZ-A-loaded pellet cores *in vitro*

The results from the *in vitro* tissue retention assay demonstrated that the percentages of remaining microspheres for CS@SZ-A@pellet cores and SA@SZ-A@pellet cores were 98.67 ± 1.22 % and 96.23 ± 1.21 %, respectively. These values were significantly higher than those of the Man@SZ-A@pellet cores, which was 40.52 ± 2.34 % (Fig. 1G and H). This finding demonstrates that the incorporation of CS and SA significantly enhances the *in vitro* adhesion of the pellet cores. This improved adhesion is expected to effectively prolong microsphere retention at colonic lesion sites, thereby improving local drug efficacy and release kinetics. This phenomenon may be attributed to the strong electrostatic interactions between the abundant amino groups (-NH_3_) in CS and the carboxyl (-COOH) and sulfate (-OSO_2_O-) groups present in mucin within the intestinal mucus layer [13]. Additionally, SA demonstrates polyanionic electrolyte properties when dissolved in water, exhibiting high viscosity upon gradual dissolution, which enhances its adhesion to the intestinal mucosa. The application of a coating layer significantly reduces the *in vitro* adhesion of the pellets, thereby protecting the pellet cores. At least a 20 % increase in total coating weight is required to ensure the unimpeded transit of coated pellets through the stomach and small intestine while preventing premature adhesion (Fig. 1I and J).

### 3.3 *In vitro* release analysis

The colon-targeted controlled release of SZ-A is essential for the effective therapeutic management of UC. In this study, the coating material employed, Eudragit® S100, is a copolymer composed of methacrylic acid and methyl methacrylate in a 1:2 ratio, which dissolves only at intestinal pH levels exceeding 7.0. The release profiles of various pellet cores were assessed after 2 h in gastric juice (Fig. 1C) or 3 h in colonic fluid (Fig. 1D), revealing that SA could moderately delay the release of SZ-A in both gastric and colonic environments, whereas CS exhibited no significant impact on SZ-A release. The coating thickness was optimized to protect the pellet cores during gastrointestinal transit and ensure their exposure at the target colonic site, thereby maximizing their adhesive potential. The release profile of SZ-A from coated pellets with different TWG was evaluated under simulated gastrointestinal conditions, including artificial gastric, intestinal, and colonic fluids at respective pH levels. The SA@SZ-A@coated pellets (Fig. 1E) and CS@SZ-A@coated pellets (Fig. 1F) were analyzed, demonstrating that the resistance of the pellets to gastric acid and small intestinal fluids increased with higher TWG of the coating. Within the same TWG of the coating, CS@SZ-A@coated pellets exhibited reduced drug release in gastric and small intestinal fluids, indicating a more robust protective effect. This phenomenon can be attributed to the decreased solubility of SA in acidic conditions and its maximal structural expansion at pH 7.4. Given that SZ-A is an alkaline drug, its interaction with water generates a localized alkaline environment within the SZ-A pellets, facilitating the dissolution and solubilization of SA, which compromises the integrity of the coatings (Fig. S3). Consequently, CS@SZ-A@coated pellets (with a 100 % TWG) demonstrated negligible drug release in gastric and small intestinal fluids, while achieving complete drug release in the colon, thereby exhibiting effective colonic targeting.

### 3.4 Kinetic mechanisms

The process of drug dissolution and release is multifaceted, and the findings indicate that both the coating and the internal structure of the pellets significantly influence drug release. In the case of uncoated pellet cores, the presence of SA modified the drug dissolution rate but did not alter the drug release mechanism, which consistently followed first-order kinetics (Tables S2–3), with Fickian diffusion governing the drug release mechanism. For coated pellets, the release time of SZ-A was prolonged, and the phase relation value was low, rendering the application of the model impractical. However, the Logistic, Hill, Boltzmann, and Gompertz models demonstrated a high degree of fit (Tables S4–5).

Of interest, the release behavior of the coated pellets in artificial colonic fluid (Fig. S4, Tables S6–7) adhered to a first-order release model. The coating layer exhibited adequate colon-responsiveness, and its thickness did not influence the rate or mechanism of drug release in colonic fluid, as anticipated, facilitating rapid localized release at the colonic site.

### 3.5 CT-based *in vitro* release

**Fig. 2:**
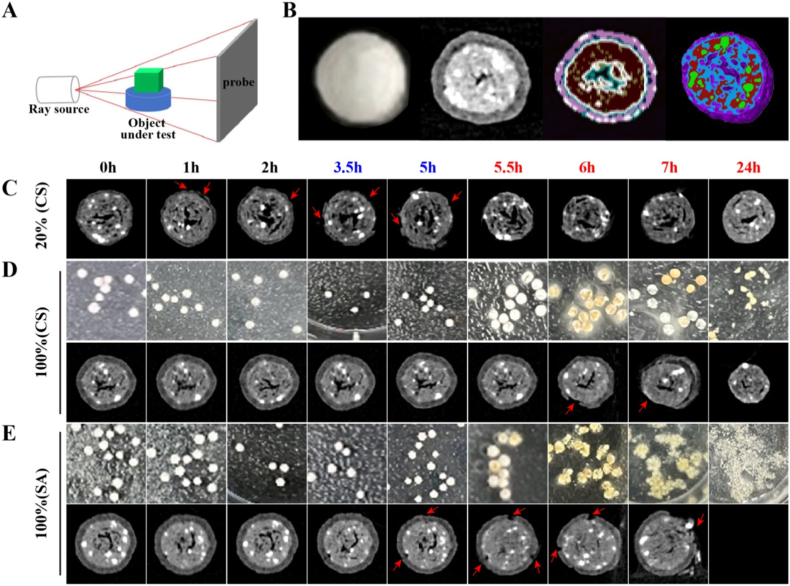
(A) Schematic diagram of the device. (B) 2D sliced, 2D rendered, and 3D rendered (differentiation of coating layer and pellet cores) images of CS@SZ-A@coated pellets with a TWG of 100 %. The samples were separately immersed in artificial gastric fluid (pH 2.0) for 2 h, after which they were removed and immersed in simulated small intestinal fluid (pH 6.8) for 3 h, followed by being placed in artificial colonic fluid (pH 7.8) for a total duration of 24 h. Internal microstructural changes of (C) CS@SZ-A@coated pellets with a 20 % TWG, (D) CS@SZ-A@coated pellets with a 100 % TWG, and (E) SA@SZ-A@coated pellets with a 100 % TWG during dissolution (the white part of the interior was calcium phosphate, and the black color was the cavities). (For interpretation of the references to color in this figure legend, the reader is referred to the Web version of this article.)

CT employs X-rays to penetrate and image the internal structure of a sample by exploiting the differential absorption of X-rays by various materials. The resulting data are processed and analyzed by a computer to generate a clear depiction of the internal structure (Fig. 2A). The CS@SZ-A@coated pellets are depicted in Fig. 2B. Two-dimensional (2D) slices revealed a cross-section of the pellet's interior, clearly distinguishing between the coating layer and the core, as well as identifying the presence of CaHPO_4_ within the core. 2D rendering and 3D rendering marked the different substances with different colors, demonstrating the thorough mixing of ingredients within the core.

The dissolution experiment based on CT was further carried out to observe the changes in the internal microstructure of the pellets during dissolution. It was observed that 20 % of the TWG of the CS@SZ-A@coated pellet cores (denoted as 20 % (CS)) was susceptible to resist gastric juice, with dissolution evident at 1 h and significant exposure of the cores to gastric juice by 2 h (Fig. 2C). This phenomenon may be attributed to the ingress of water through the pores of the coating layer, which facilitated the migration of the water-soluble drug to the exterior, thereby accelerating the rupture of the coating layer. Conversely, the CS@SZ-A@coated pellets of a 100 % TWG (noted as 100 % (CS)) maintained the integrity of the coating layer in both gastric and small intestinal fluids. These pellets gradually dissolved at the pH level of colonic fluids, with the intact shedding of the coating layer observed in some pellets at 2 h in colonic fluids (7 h of the experiment) (Fig. 2D). The SA@SZ-A@coated pellets of a 100 % TWG (designated as 100 % (SA)) exhibited a structural degradation of the coating layer observable at 5 h, corroborating the *in vitro* release findings. Water infiltration into the pellet core dissolved SZ-A, creating a localized alkaline environment that induced SA swelling. The swollen SA filled the CT-visible voids (appearing black), thereby generating internal stress that caused premature coating rupture (the mechanism was validated in Fig. S3). Concurrently, the spherical morphology progressively degraded as the SA dissolved (Fig. 2E).

### 3.6 Distribution and form of drugs

**Fig. 3:**
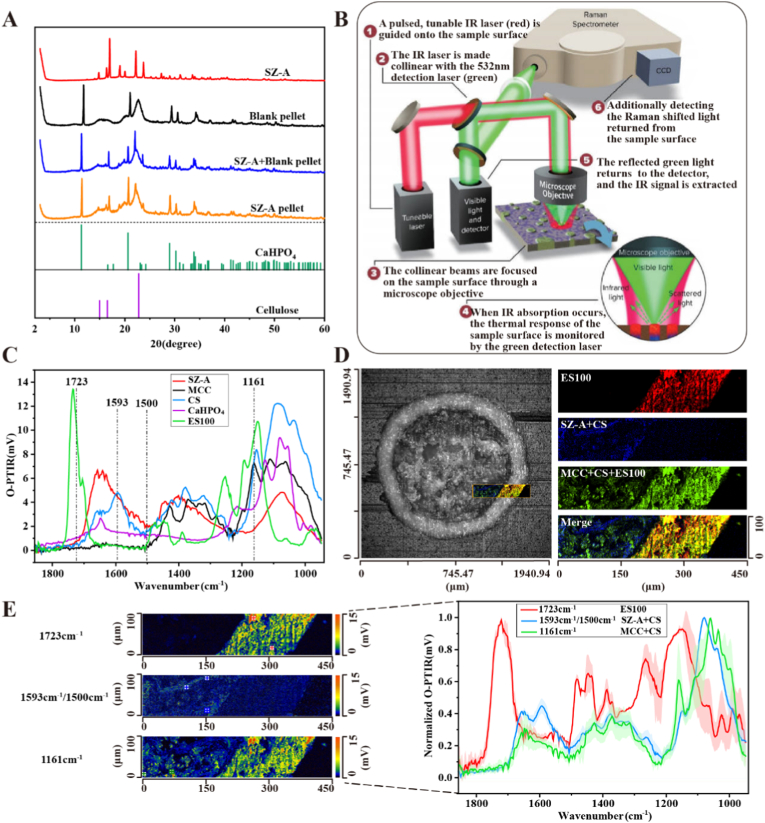
Distribution and presence of drugs. (A) XRD analysis. The distribution of components in CS@SZ-A@coated pellets was studied by O-PTIR. (B) Instrument and principle. mIRage™ O-PTIR works by focusing a single pulsed infrared laser (red) and a visible detection laser (green) on the surface of the sample at the same time. When IR absorption occurs, the thermal response on the surface of the material is detected by the green detection laser. The signal extracted by the detector can be used to assess the chemical composition of the particles [15]. The distribution of different materials can be distinguished by detecting the strength of the response signal of the sample cross-section at different specific infrared wavelengths. Modified and copied with the permission of Quantum Design. (C) O-PTIR mapping of each component for determining the characteristic wavelength of a single component. (D) Optical and overlay images of the cross-section of the pellet showed that the components were uniformly distributed in the pellet. (E) Chemical imaging of the pellet in specific wavelength bands and precise spectra at different locations (red, blue, and green dots) to confirm the presence of the substance by characteristic peaks. (For interpretation of the references to color in this figure legend, the reader is referred to the Web version of this article.)

The XRD analysis (Fig. 3A) indicated that the positions, shapes, and relative intensities of the characteristic peaks of the drug-loaded pellets (SZ-A pellets) remained unchanged, as did those in the physical mixtures of the drug and blank pellets (SZ-A + Blank pellet). These results demonstrate that the pellet preparation process did not induce any significant alterations in the crystal structure of SZ-A, confirming the stability of the pellet formulation.

In this study, O-PTIR was employed for the first time to assess the distribution of components within CS@SZ-A@coated pellets (Fig. 3) and SA@SZ-A@coated pellets (Fig. S5). This technique offers the advantage of not requiring fluorescent markers and provides high spatial resolution (∼300 nm) [14], facilitating a more intuitive and comprehensive understanding of the chemical information present in the minute regions of the sample surface at the submicron scale (Fig. 3B). Fig. 3C illustrates the characteristic excitation wavelengths corresponding to each component within the CS@SZ-A@coated pellets. Representative regions within its cross-section were selected for imaging, and specific photothermal response profiles were obtained at 1723 cm^−1^, 1593 cm^−1^/1500 cm^−1^ (utilizing a ratio to mitigate interference from thermal effects), and 1161 cm^−1^, respectively (Fig. 3E). Three randomly selected points were scanned independently to acquire infrared spectra, and error bands (red, blue, and green) were plotted to confirm the presence of the substance. The results indicated that the response signals were uniformly distributed across the scanning region at specific wavelengths, and the narrow error bands of the IR spectra demonstrated that the components were uniformly mixed. The components were assigned pseudo-colors, and their distribution is illustrated in Fig. 2D. Eudragit® S100 exhibited a uniform distribution within the coating layer and did not migrate into or penetrate the pellet cores. The mixtures of SZ-A and CS, as well as MCC and CS, were uniformly distributed within the pellet cores. Collectively, these findings confirm the effective encapsulation of SZ-A within the pellets and the uniform distribution of the components.

### 3.7 *In vivo* targeting and retention of SZ-A-coated pellets in the colon

**Fig. 4:**
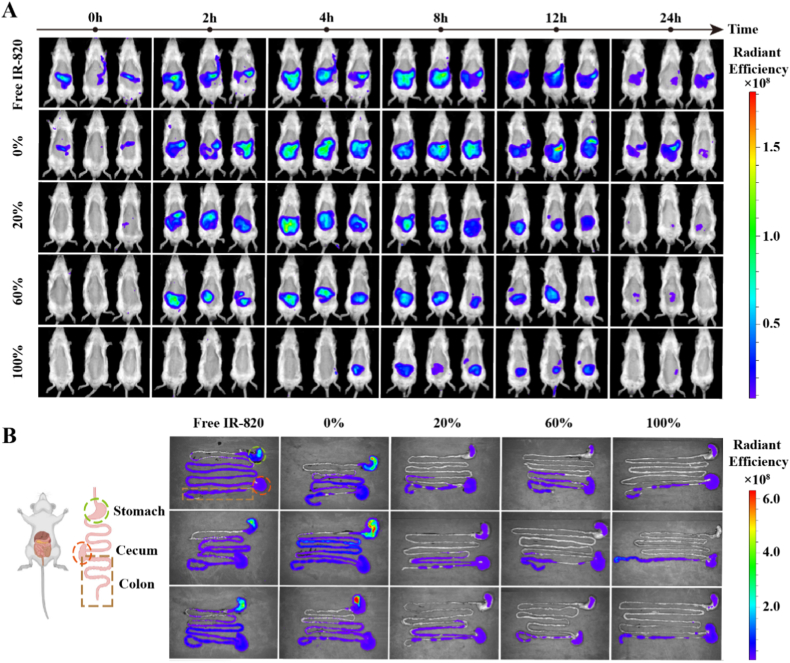
*In vivo* biological distribution of colon-targeting pellets (n = 3), coated pellets with a TWG of 100 % showed colon-targeting and retention. (A) Fluorescent images of SD rats at 0, 2, 4, 8, 12, and 24 h after oral administration of pellet cores and coated pellets. A 100 % TWG resists the erosion of gastric and small intestinal fluids, which release drugs when they reach the colon. (B) Fluorescent images of the final endpoint gastrointestinal tract.

The results from *in vivo* imaging of small animals are presented in Fig. 4A. Compared with the Free IR-820 group, the 0 % group maintained strong fluorescence intensity at 24 h, with fluorescence signals predominantly localized in the gastrointestinal tract. This suggests that the pellet cores have an extended retention time *in vivo*, potentially due to the bioadhesive properties of CS. The appearance of the fluorescent signal was delayed with increasing TWG (coating thickness) of the coated pellets. In the 100 % group, weak fluorescence was not observed until 4 h, indicating that the coating layer resists erosion by gastric and small intestinal fluids and exhibits some colonic targeting properties. The generally weak fluorescence signal observed in this group may be attributed to the incomplete dislodgement of the pellet coating layer, which hindered the complete release of the poorly water-soluble fluorescent dyes. The fluorescence distribution within the gastrointestinal tract at the endpoint (Fig. 4B) revealed fluorescence residues throughout the entire gastrointestinal tract in the Free IR-820 group at 24 h. In the 0 % coating group, fluorescence was mainly distributed in the stomach, which confirmed the previous speculation. In the 20 % and 60 % coating groups, IR-820 leakage was observed in the small intestine, attributed to inadequate coating thickness. Conversely, in the 100 % coating group, fluorescence was primarily concentrated in the colon and cecum, indicating effective colon targeting. This finding was consistent across the qualitative assessment (Fig. S6).

In conclusion, the coated pellets with a TWG of 100 % demonstrated resistance to gastric and small intestinal fluids. Upon reaching the colon, the coating layer began to dissolve under colonic pH conditions, facilitating the release of the drug and indicating a degree of colonic targeting. The detachment of the coating enhanced the adhesive properties of the pellet cores, potentially increasing the retention time of the pellets within the body and thereby optimizing the drug's therapeutic efficacy.

### 3.8 Tissue distribution based on AFADESI-MSI

**Fig. 5:**
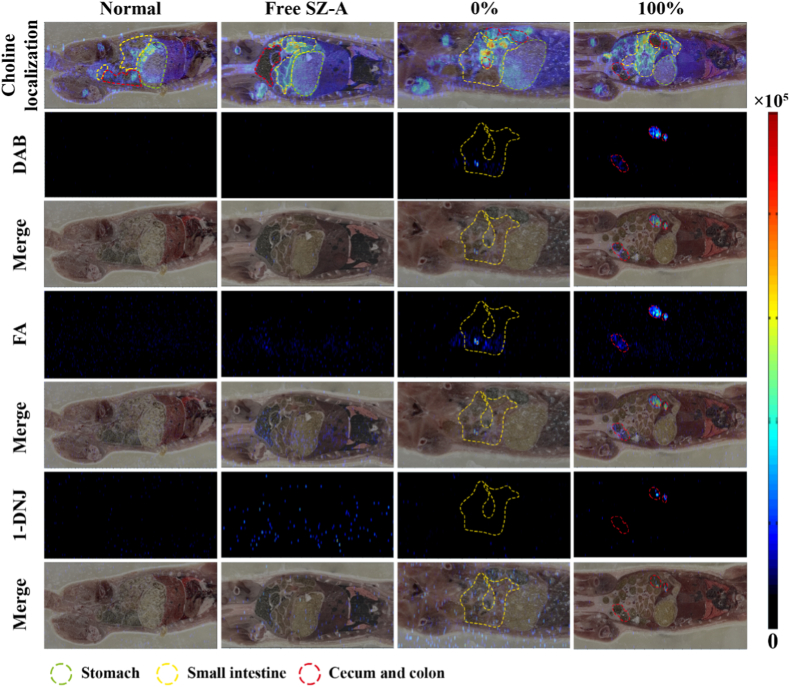
Distribution of the three main components of SZ-A, DAB, FA, and 1-DNJ, in rat tissues.

AFADESI-MSI was employed to characterize the *in vivo* distribution of the three primary components of SZ-A: DAB, FA, and 1-DNJ. The hydrogenated ion images of these molecules are presented in Fig. 5, with *m*/*z* values of 134.0811 ± 0.005 for DAB, 148.0968 ± 0.005 for FA, and 164.0917 ± 0.005 for 1-DNJ. The findings demonstrated that the free SZ-A solution (referred to as Free SZ-A) underwent metabolism 6 h post-gavage, with no detectable signals of the three molecules in the rat tissue sections. In contrast, 6 h following the administration of CS@SZ-A@pellet cores (denoted as 0 %), SZ-A was predominantly localized in the small intestine. The pellet cores facilitated an extended *in vivo* retention time compared to the free solution, thereby highlighting the adhesive properties of the pellet cores.

CS@SZ-A@coated pellets (denoted as 100 %) demonstrated a predominant distribution of the SZ-A signal within the colonic region at 6 h post-administration. These findings indicate that the coating layer effectively facilitated the transport of the pellets to the colon. The drug was released after reaching the colon, reflecting the colon's targeting characteristics. Furthermore, the adhesive properties of the pellet cores are likely to enhance their retention time within the colon, which would promote the full release of the drug and exert its efficacy.

### 3.9 *In vivo* therapeutic efficacy on TNBS-induced colitis

**Fig. 6:**
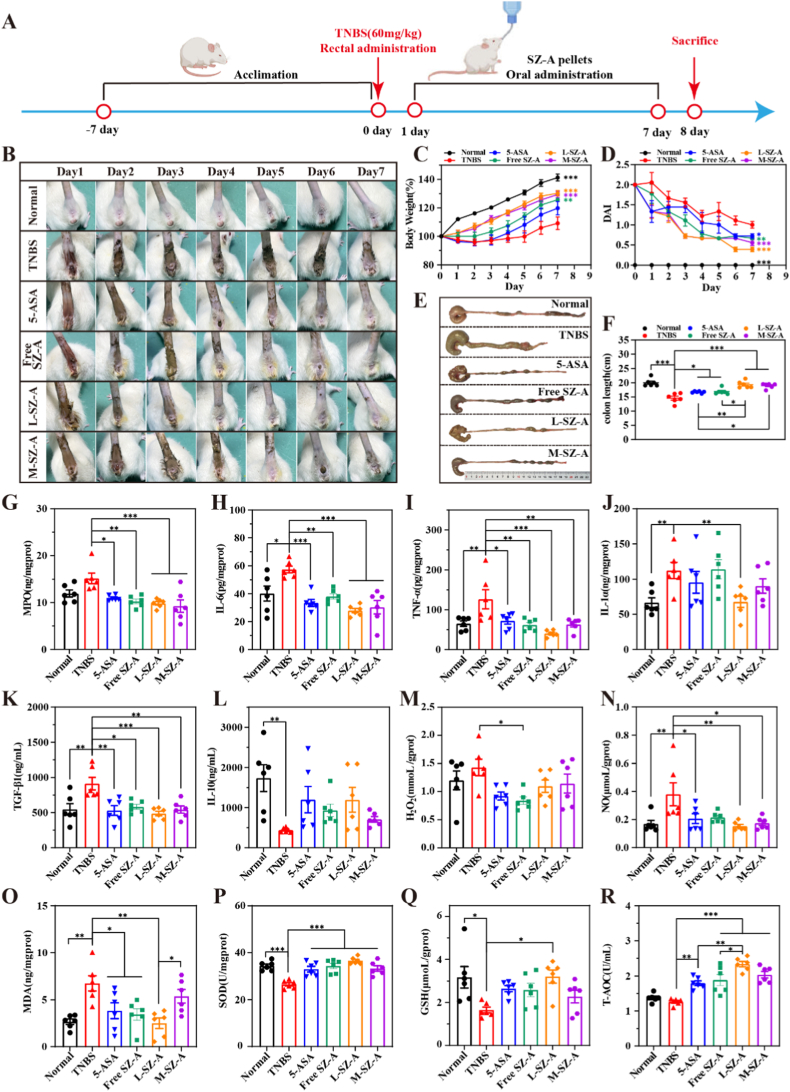
CS@SZ-A@coated pellets showed a strong curative effect on the rat colitis model. (A) The UC model was constructed by rectal injection of TNBS-ethanol solution in SD rats. Rats were gavaged once daily with 5-ASA (100 mg/kg), free SZ-A solution (Free SZ-A, SZ-A 34.62 mg/kg), low-dose CS@SZ-A@coated pellets (L-SZ-A, SZ-A 17.31 mg/kg), or medium-dose CS@SZ-A@coated pellets (M-SZ-A, SZ-A 34.62 mg/kg). (B) Images of representative rat fecal conditions. (C) Graphs of rat body weight change over time, normalized to percentage of initial body weight for the 6 intervention modalities. (D) Plot of changes in rat DAI score. (E) Representative colon images of rats in each group. (F) Statistics of colon length in different groups. (G–L) Histogram analysis of MPO, IL-6, TNF-α, IL-1α in colon tissue and TGF-β1 and IL-10 in serum in 6 groups. (M–R) Histogram analysis of changes in oxidative stress markers (H_2_O_2_, NO, MDA, SOD, GSH, T-AOC) in colonic tissues. Data were expressed as mean ± S.E.M. (n = 6) and analyzed by one-way ANOVA: ∗p < 0.05, ∗∗p < 0.01, ∗∗∗p < 0.001.

The TNBS model (Th1/Th17-driven) was used to establish a detailed dose-response. In the TNBS-induced colitis model, ethanol effectively compromised the integrity of the intestinal barrier, facilitating the interaction of the semi-antigenic reagent TNBS with colonic histones [16], thereby activating the immune system and triggering an inflammatory response. The intervention protocol is illustrated in Fig. 6A. We conducted a direct comparison of the efficacy of CS@SZ-A@coated pellets with the commonly used clinical therapeutic agent 5-aminosalicylic acid (5-ASA) and a free SZ-A solution. Observations of blood in the stool, depressive behavior, significantly diminished vigor, unformed feces, and decreased body weight in rats one day post-modeling (day 1 of the experiment) confirmed the successful establishment of the model. Within 1 week, the TNBS group exhibited persistent hemorrhagic diarrhea (Fig. 6B), significant weight loss, piloerection, and reduced locomotor activity. As depicted in Fig. 6C, UC rats in the L-SZ-A and M-SZ-A groups demonstrated significantly greater body weight gain compared to the TNBS group, with effects surpassing those observed in the Free SZ-A and 5-ASA groups. A similar trend is evident in Fig. 6B.

Inflammation can result in the atrophy and fibrosis of colon tissue, consequently reducing the colon's length. Statistical analysis of colon length revealed that the colon in the L-SZ-A group was significantly longer than that in the TNBS group and comparable to that in the Normal group. Furthermore, the colon length in the L-SZ-A group exceeded that of other treatment groups (Fig. 6E and F). These findings suggest that L-SZ-A effectively mitigates TNBS-induced colon atrophy. Notably, L-SZ-A demonstrated greater efficacy in alleviating colitis symptoms compared to the conventionally used UC therapy, 5-ASA.

Notably, the L-SZ-A group demonstrated efficacy comparable to or even superior to both the M-SZ-A group and the Free SZ-A group, revealing a non-linear dose-response relationship. This phenomenon suggests that for colon-targeted delivery systems, beyond the total administered dose, both local drug exposure characteristics and release kinetics are crucial determinants of therapeutic outcome. This non-linear relationship indicates the potential existence of an optimal therapeutic window for colon-targeted drug delivery, wherein excessive local drug concentrations or systemic exposure may not significantly enhance treatment efficacy. The low-dose CS@SZ-A@coated pellets maintain appropriate drug concentrations at the colonic site, enabling full therapeutic action while avoiding potential saturation effects or counter-regulatory responses. Through its “golden cicada-escape” release mechanism, the formulation achieves sustained drug delivery to the colonic mucosa. Compared to the higher local concentrations of M-SZ-A or the systemic exposure from the free SZ-A solution, this sustained-release profile provides a more optimized pharmacokinetic foundation for long-term processes such as epithelial repair and immune modulation in UC.

### 3.10 Anti-inflammatory activities and antioxidant properties of CS@SZ-A@coated pellets

Inflammatory mediators, including interleukin-1 (IL-1), interleukin-6 (IL-6), tumor necrosis factor-α (TNF-α), interferon-γ (IFN-γ), and chemokine (C-C motif) ligand 2 (CCL2), along with excessive reactive oxygen species (ROS) and reactive nitrogen species (RNS), are intricately linked to the pathogenesis of UC c), [17]. In the context of UC, the intestinal epithelium is subjected to substantial levels of inflammatory cytokines produced by activated immune cells. These cytokines compromise the epithelial barrier function, either directly or indirectly, and exacerbate tissue damage through various mechanisms, including the induction of leukocyte recruitment, activation, and migration; the release of proteases from activated neutrophils; increased apoptosis of epithelial cells; and disruption of the *in vivo* balance of cellular proliferation, differentiation, and apoptosis [3a]. MPO, secreted by activated neutrophils, serves as a biomarker for assessing the extent of inflammation [18]. The concentrations of MPO and inflammatory mediators were quantified using ELISA to assess the anti-inflammatory efficacy of CS@SZ-A@coated pellets. As shown in Fig. 6G, TNBS treatment resulted in significantly elevated MPO levels in the colonic tissues, whereas the lowest MPO levels were observed after CS@SZ-A@coated pellets treatment compared with the 5-ASA or Free SZ-A groups. The expression of IL-6, TNF-α, IL-1α, and transforming growth factor-β1(TGF-β1) was all significantly elevated in the TNBS group, and the expression of IL-10 was significantly decreased, whereas the L-SZ-A group significantly decreased the expression of pro-inflammatory cytokines, while increasing the expression of anti-inflammatory factors (Fig. 6H–L). Notably, the levels of these pro-inflammatory cytokines were also lower in the L-SZ-A and M-SZ-A groups compared with the 5-ASA group and the Free SZ-A group, and collectively, the L-SZ-A group was more advantageous than the M-SZ-A group, which suggests that L-SZ-A is more effective in suppressing TNBS-induced inflammatory responses.

ROS in UC can cause mucosal damage, maintain a pro-inflammatory microenvironment, and dysregulate the intestinal microbiota [19]. Compared with the Normal group, the levels of H_2_O_2_, NO, and the oxidative stress product MDA in the colon of TNBS-induced rats were significantly increased (Fig. 6M–Q). Oral administration of L-SZ-A significantly reduced the levels of NO and MDA in rats, which were lower than those in the 5-ASA, Free SZ-A, and M-SZ-A groups. The activity of the antioxidant enzyme SOD and the content of non-enzymatic antioxidant GSH were decreased in the TNBS group but increased in the L-SZ-A group. T-AOC (Fig. 6R) can indicate the antioxidant capacity of the organism. Among all the treatment groups, the therapeutic effect of CS@SZ-A@coated pellets was the best, significantly enhancing the antioxidant capacity. Interestingly, the L-SZ-A group showed a better therapeutic effect than the M-SZ-A group, and there were significant differences compared with the 5-ASA group and the Free SZ-A group. The advantages of colon-targeted formulation were fully demonstrated.

### 3.11 Restoration of colonic barrier

**Fig. 7:**
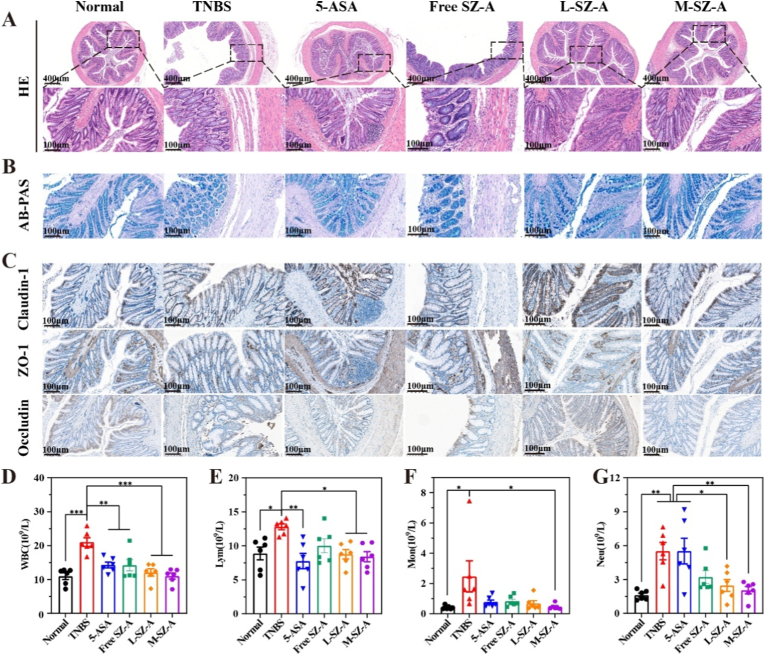
Colonic barrier recovery in TNBS-induced UC rats after CS@SZ-A@coated pellets treatment. (A) Representative sections of H&E-stained colon tissues from different treatment groups; (B) AB/PAS staining; (C) Immunohistochemical staining of TJ proteins Claudin-1, ZO-1, and Occludin. Scale bars, 400 μm, 100 μm. The number of (D)white blood cells, (E)neutrophils, (F)lymphocytes, and (G)monocytes in the blood. The data were expressed as mean ± S.E.M. (n = 6), and were significant by one-way ANOVA: ∗p < 0.05, ∗∗p < 0.01, ∗∗∗p < 0.001.

The colonic barrier is characterized by a complex composition, encompassing a chemical barrier, a mechanical barrier (also referred to as a physical barrier), an immune barrier, and a biological barrier. To substantiate the therapeutic efficacy, H&E staining of colonic tissue was conducted. In the TNBS-induced rat model of colitis, a characteristic pattern of colitis was observed, including structural destruction of epithelial cells, loss of crypts, and infiltration of inflammatory cells. As illustrated in Fig. 7A, the crypts appeared more intact, and there was a reduction in inflammatory cell infiltration in the L-SZ-A group, indicating that low-dose CS@SZ-A@coated pellets effectively mitigated the symptoms of colonic inflammation.

The mucus layer, composed of mucin secreted by goblet cells, serves as the body's primary defense against damage to intestinal mucosal tissues from the complex external environment and is classified as part of the chemical barrier. It has been demonstrated that UC is associated with a diminished secretory response of goblet cells [20]. In rat colonic tissues, a significant reduction in the number of goblet cells and the thickness of the mucus layer was observed following TNBS induction, whereas intervention with the L-SZ-A group ameliorated these deficits (Fig. 7B).

Tight junctions (TJs) between epithelial cells are critical components of the intestinal mucosal mechanical barrier, serving to restrict the entry of pathogens and foodborne antigens while permitting the selective absorption of nutrients and water [21]. Zonula occludens 1 (ZO-1) was the first TJ protein identified, functioning as a link between TJ transmembrane proteins and the actin cytoskeleton, and playing a crucial role in regulating TJ function. Occludin and Claudin are both transmembrane proteins; a decrease in Occludin expression is associated with increased permeability of the leaky pathway observed in UC. Based on their effect on epithelial permeability, Claudins are categorized into dense (1, 3, 4, 5, and 18), which enhance barrier tightness, and leaky (2, 10, and 15), which increase paracellular permeability [2]. Immunohistochemical analysis revealed a significant reduction in the expression of Claudin-1, Occludin, and ZO-1 in the colonic tissue of the TNBS-treated group (Fig. 7C), suggesting a substantial impairment of epithelial barrier function due to TNBS treatment. In contrast, the L-SZ-A group demonstrated normalized expression levels of these proteins in the rat colon.

Additionally, the L-SZ-A group exhibited a reduction in the counts of various immune cell types, including white blood cells (WBC), neutrophils (Neu), lymphocytes (Lym), and monocytes (Mon) (Fig. 7D–G). These findings indicate that L-SZ-A may effectively attenuate the activation of immune cells implicated in the pathogenesis of colitis [3a].

### 3.12 Modulation of gut microbiome

**Fig. 8:**
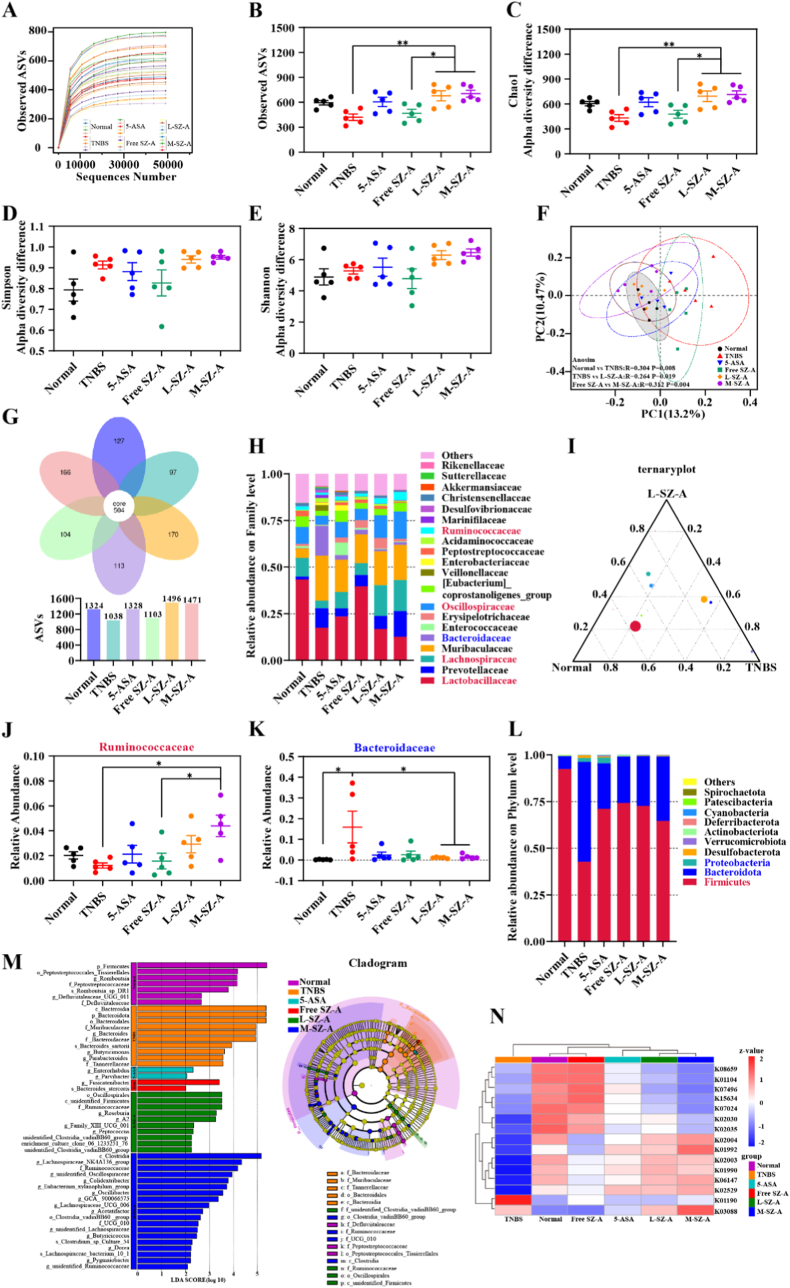
CS@SZ-A@coated pellets alter the composition of the gut microbiota. SD rats were given TNBS-ethanol rectum, and the treatment method is shown in Fig. 5A. Stool collected on day 8 was analyzed by 16S rDNA sequencing for gut microbiome analysis. (A) Dilution curves for each sample. α-diversity (B) observed ASV number and (C) Chao1 index. β-diversity (D) Simpson Index and (E) Shannon Index. (F) PCoA based on unweighted Unifrac distance. Similarity analysis (ANOSIM) was used to determine the statistical significance of intestinal flora composition, R > 0, indicating differences between groups, P < 0.05, 0.01, or within the range of both, indicating significant differences between groups. Normal *vs.* TNBS R = 0.304, P = 0.008; TNBS *vs.* L-SZ-A R = 0.264, P = 0.019; Free SZ-A *vs.* M-SZ-A R = 0.312, P = 0.004. (G) Petal maps reflecting the common and unique ASVs of each group. (H) Histogram of relative species abundance at the family level. Red indicated families with increased relative abundance after treatment, and blue indicated families with decreased relative abundance after treatment. (I) ternary phase diagram of intestinal flora in the Normal group, TNBS group, and L-SZ-A group at the family level. Relative abundances of (J) Bacteroidaceae and (K) Ruminococcaceae in each group. (L) Phylum-level species relative abundance histogram. Red represented the phyla with increased relative abundance after treatment, and blue represented the phyla with decreased relative abundance after treatment. (M) Histogram of the distribution of LDA values at the phyla to species level (left) and phyla to family level (right). (N) Functional prediction based on the KO database. Data are expressed as mean ± S.E.M. (n = 5). Significance analyzed by one-way ANOVA: ∗p < 0.05, ∗∗p < 0.01, ∗∗∗p < 0.001. (For interpretation of the references to color in this figure legend, the reader is referred to the Web version of this article.)

The intestinal mucosal biological barrier is predominantly influenced by a diverse array of microbial species and their metabolic products, with the preservation of this functional equilibrium being crucial for sustaining normal mucosal physiology [22]. Numerous studies have demonstrated that the onset of UC is associated with a dysbiosis of the intestinal microbiota [23] and that a therapeutic strategy combining inflammation suppression with microbiota management can enhance UC treatment efficacy. Consequently, we further explored the potential of low-dose CS@SZ-A@coated pellets treatment to modulate the intestinal microbial composition in TNBS-induced colitis in rats. We conducted sequencing and analysis of the V4 region of the 16S ribosomal RNA gene from fecal samples. Raw reads were screened and assembled, and representative amplicon sequence variants (ASVs) were identified through clustering at 100 % similarity. Utilizing the ASVs, we conducted both α and β diversity analyses. The species diversity curves (Fig. 8A) indicated that the sequencing data were sufficiently comprehensive to capture the majority of microbial diversity present in the samples.

The L-SZ-A group demonstrated a significant enhancement in bacterial abundance, as indicated by the Chao1 index and the number of observed ASVs, in TNBS-induced colitis rats (Fig. 8B and C). This increase was comparable to the levels observed in healthy rats and surpassed the effects observed in the Free SZ-A group, highlighting the formulation's advantages. The petal plot (Fig. 8G) demonstrated the same results. However, there were no significant changes in species diversity, as measured by the Shannon and Simpson indices (Fig. 8D and E).

Principal Coordinate Analysis (PCoA) revealed that the gut microbiota profile of the TNBS group differed from that of the Normal group (Anosim analysis, P = 0.008, R = 0.304). Different treatment groups exerted distinct regulatory effects on gut microbiota, with the L-SZ-A group showing a significant divergence from the TNBS group (Anosim analysis, P = 0.019, R = 0.264), and the confidence intervals overlapped with those of the Normal group, indicating similar gut microbiota characteristics (Fig. 8F). These findings suggest that low-dose CS@SZ-A@coated pellets can effectively reverse intestinal flora dysbiosis and exert significant therapeutic effects on colitis.

Analysis of the gut microbiota composition demonstrated that TNBS administration induced significant structural changes in the microbial communities, spanning from broad phylum-level classifications to finer genus-level resolutions, relative to the normal control group (phylum, Fig. 8L; class, Fig. S8A; family, Fig. 8H; genus, Fig. S10A). Further analysis at the phyla/class level revealed that the L-SZ-A group significantly increased the relative abundance of *Firmicutes* (Fig. S7A), especially *Clostridia* (Fig. S8B), which are known to promote the accumulation of T regulatory cells and support immune homeostasis [24]. And there was a decrease in the relative abundance of *Bacteroidota* (Fig. S7B) and *Proteobacteria* (Fig. S7C), particularly in the *Bacteroidia* (Fig. S8C) and *Gammaproteobacteria* (Fig. S8D) classes. At the family level, the L-SZ-A group significantly increased the relative abundance of beneficial bacteria such as *Lactobacillaceae* (Fig. S9A, known to be beneficial to UC animal models and patients [25]), *Lachnospiraceae* (Fig. S9B, which produces butyrate and enhances epithelial barrier integrity and inhibits inflammation [26]), as well as *Oscillospiraceae* (Fig. S9C) and *Ruminococcaceae* (Fig. 8J). In contrast, the relative abundance of *Bacteroidaceae* (Fig. 8K) was reduced. The ternary phase diagram further depicted the distribution proportions of different species across the Normal, TNBS, and L-SZ-A groups (Fig. 8I). Furthermore, at the genus level, the relative abundance of intestinal pathogenic or potentially pathogenic microorganisms, including *Bacteroides* (Fig. S10C), *Escherichia-Shigella* (Fig. S10D) [27], and *Prevotella* (Fig. S10E, known to exacerbate intestinal inflammation and increase intestinal permeability [28]), decreased following treatment. In contrast, the relative abundance of *Lactobacillus* (Fig. S10B) exhibited an increasing trend.

Additionally, Linear Discriminant Analysis Effect Size (LEfSe) was employed to identify biomarkers exhibiting statistically significant differences across various taxonomic levels, ranging from phylum to species (log [LDA] > 2). Fig. 8M distinctly illustrated that Firmicutes constituted the predominant flora in the normal group. Conversely, following TNBS treatment, Bacteroidia emerged as the primary component of the colonic flora. After various treatments, Oscillospirales, Clostridia, and other probiotics became dominant, corroborating previous findings regarding species abundance.

PICRUSt2 was employed to predict the metabolic pathways and functions of gut microbiota. The KEGG ORTHOLOGY (KO)-based functional relative abundance clustering heatmap is presented in Fig. 8N, where functions with similar compositions and similar groups are clustered together. By comparing the composition of the same function across different samples, it was observed that the abundance of ATP-binding cassette transporter (ABC transporter) components—specifically K06147, K02003, K01990, K02004, K01992, K02030, and K02035, which are involved in the transport of nutrients such as amino acids and the expulsion of harmful substances such as secondary metabolites [29]—was reduced in the TNBS group compared to the normal group. Additionally, the abundance of protein tyrosine phosphatase [30] K01104, which contributes to the integrity of the intestinal epithelial barrier, and the key enzyme [31] K15634, which is involved in carbohydrate degradation, was also decreased in the TNBS group. Conversely, the abundance of the energy coupling factor (ECF) subfamily RNA polymerase sigma-70 factor [32] K03088, which regulates the expression of virulence genes, was increased in the TNBS group.

In summary, TNBS partially disrupted the metabolic function of the gut microbiota, subsequently compromising the integrity of the colonic barrier. Analysis via 16S rDNA sequencing revealed that L-SZ-A effectively optimized the composition of the intestinal microbiota, significantly enhancing the abundance of probiotic species while suppressing pathogenic flora, thereby promoting intestinal homeostasis. This effect may be attributed to its ability to restore enterobacterial metabolism and facilitate the repair of the colonic mucosal barrier.

### 3.13 Amelioration of DSS-induced colitis

**Fig. 9:**
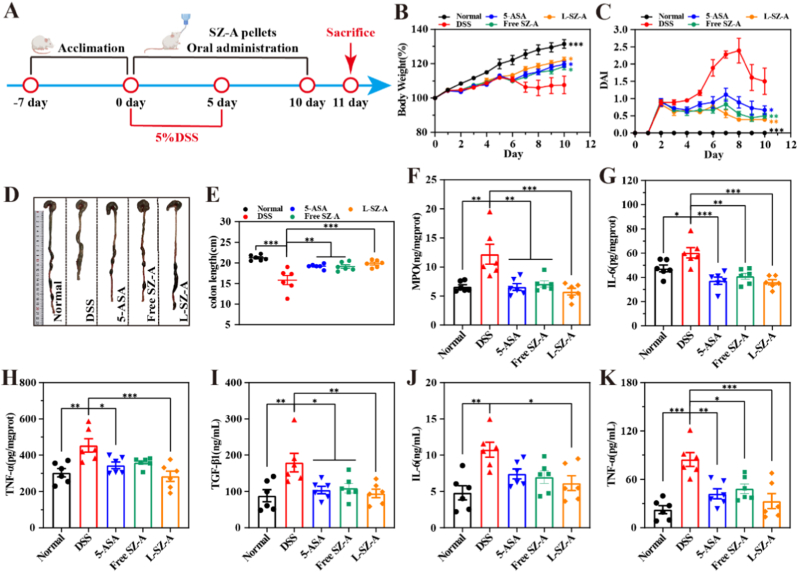
Low-dose CS@SZ-A@coated pellets relieved DSS-induced colitis. (A) Dosing regimen, (B) Daily weight change, (C) DAI score, (D) Representative colon images, and (E) colon length. (F) MPO content and (G) IL-6, (H) TNF-α concentration in colon tissue. Serum concentrations of (I) TGF-β1, (J) IL-6, and (K) TNF-α. The data were expressed as mean ± S.E.M. (n = 6), and were significant by one-way ANOVA: ∗p < 0.05, ∗∗p < 0.01, ∗∗∗p < 0.001.

The DSS-induced rat model of UC effectively recapitulates key pathological features of human disease. Based on previous findings demonstrating the superior efficacy of low-dose CS@SZ-A@coated pellets in the TNBS-induced (immune-driven) colitis model, we selected this optimal dose to evaluate therapeutic efficacy in the DSS-induced (epithelial injury-driven) model, thereby systematically assessing the formulation's potential across distinct pathogenic mechanisms. Post-induction, the rats exhibited symptoms such as mucus-like stools, fecal occult blood, reduced colon length, weight loss, decreased mobility, poor coat condition, inflammatory cell infiltration, and colonic epithelial damage. Following the initiation of treatment, rats in the DSS group began to regain body weight, with a significant difference observed between the body weights of the L-SZ-A group and the DSS group from the 7th day of treatment. By the tenth day of L-SZ-A treatment, the animals were effectively protected against DSS-induced increases in DAI, colon shortening, and elevated MPO content (Fig. 9A–F). Moreover, L-SZ-A treatment suppressed colonic and serum levels of IL-6 and TNF-α, and inhibited serum TGF-β1 levels (Fig. 9G–K).

**Fig. 10:**
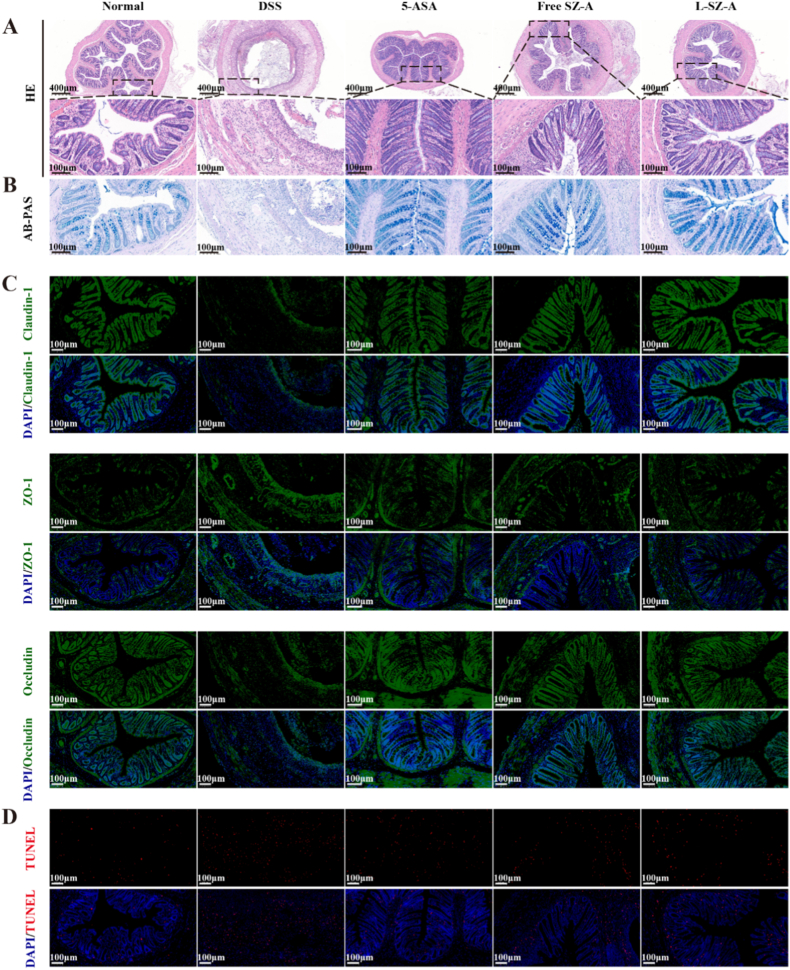
Low-dose CS@SZ-A@coated pellets repaired the colonic barrier in DSS-induced colitis. (A) Representative sections of H&E-stained colon tissues from different treatment groups; (B) AB/PAS staining; (C) IF staining of TJ proteins Claudin-1, ZO-1, and Occludin. Scale bars, 400 μm, 100 μm. (D) TUNEL staining.

In the DSS model, there was a notable depletion of the mucus layer, a reduction in goblet cell numbers, and a diminished expression of key TJ proteins, specifically Claudin-1 and occludin. In the L-SZ-A group, there was a significant increase in goblet cell numbers and an upregulation of TJ-associated protein expression in the colon. This intervention effectively safeguarded the epithelial cells from apoptosis and reinstated the integrity of the intestinal barrier (Fig. 10A–D).

### 3.14 Safety assessment

**Fig. 11:**
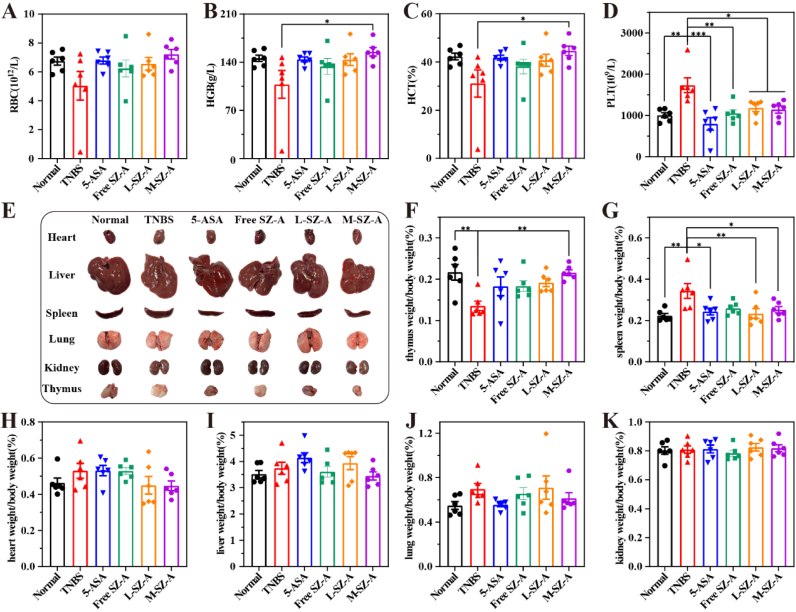
Number of (A) red blood cells, (B) hemoglobin content, (C) red blood cell pressure-volume, and (D) platelet count. (E) Representative major organ pictures and organ indices (F) Thymus index (G) Spleen index (H) Heart index (I) Liver index (J) Lung index (K) Kidney index. The data were expressed as mean ± S.E.M. (n = 6), and were significant by one-way ANOVA: ∗p < 0.05, ∗∗p < 0.01, ∗∗∗p < 0.001. (For interpretation of the references to color in this figure legend, the reader is referred to the Web version of this article.)

The hematological analysis revealed a reduction in red blood cell count, hemoglobin concentration, and mean corpuscular volume in rats subjected to TNBS induction, while the L-SZ-A treatment group demonstrated a normalization of these parameters (Fig. 11A–D). Histopathological examination showed no significant lesions in the major organs across the groups (Fig. 11E). Relative to the Normal group, TNBS exposure resulted in an increased splenic index and a decreased thymic index (Fig. 11F and G), potentially linked to the inflammatory response and immune status. The indices for the heart, liver, spleen, lung, kidney, and thymus in the treatment group did not differ significantly from those in the Normal group (Fig. 11F–K). Serum biochemical analysis in the DSS-induced model further validated the treatment's safety profile. The results showed that the key hepatic (AST, ALT), renal (urea, Cr), or cardiac (CK, LDH) function parameters remained within normal ranges (Fig. S11). The consistent safety profile observed across two distinct colitis models supports the favorable safety characteristics of the CS@SZ-A@coated pellets.

## 4 Conclusion

This study developed CS@SZ-A@coated pellets as a colon-targeted pH-responsive system for effective delivery of water-soluble multicomponent small-molecule drugs in UC treatment. The formulation demonstrates gastric resistance and achieves site-specific adhesion and drug release through a “golden cicada-escape” mechanism, showing enhanced therapeutic efficacy with reduced systemic effects. Its translational potential is supported by proven and reliable manufacturing processes and maintained physical properties and structural integrity under ambient conditions, while its bioactivity has been validated in preventive models of different pathological mechanisms (immune-mediated and epithelial injury models).

Several limitations should be acknowledged, particularly the restricted availability of genetically engineered rat strains and species-specific reagents for rats compared to murine systems, which constrains detailed mechanistic investigation. Furthermore, validation in therapeutic intervention models would strengthen clinical relevance. Future work will focus on advancing formulation strategies through techniques like hot-melt extrusion and elucidating SZ-A's molecular mechanisms, particularly its regulation of upstream signaling pathways such as NF-κB and Nrf2. These complementary approaches will accelerate both technological innovation and mechanistic understanding in UC therapeutics.

## CRediT authorship contribution statement

**Zihan Liu:** Writing – original draft, Validation, Methodology, Investigation. **Shan Chen:** Writing – review & editing, Resources. **Jinghan Yu:** Validation, Investigation. **Zhiyang Wen:** Validation, Investigation. **Yue Gao:** Validation, Investigation. **Yanfang Yang:** Writing – review & editing. **Hongliang Wang:** Writing – review & editing, Supervision. **Qingce Zang:** Writing – review & editing, Methodology. **Jun Ye:** Writing – review & editing, Supervision, Funding acquisition, Conceptualization. **Yuling Liu:** Writing – review & editing, Supervision, Conceptualization.

## Declaration of competing interest

The authors declare that they have no known competing financial interests or personal relationships that could have appeared to influence the work reported in this paper.

## Data Availability

Data will be made available on request.
