# Supplementary material for: “Golden cicada-escape” style colon-targeted pellets for ulcerative colitis by balancing oxidative stress and repairing colonic barrier
Source: Mater Today Bio. 2025 Nov 25;35:102608. doi: 10.1016/j.mtbio.2025.102608 (PMC12702123; doi:10.1016/j.mtbio.2025.102608)
Supplement: Multimedia component 1 [file mmc1.docx]

**Supplemental Materials**

**1. Preparation and characterization of CS@SZ-A@coated pellets**

**
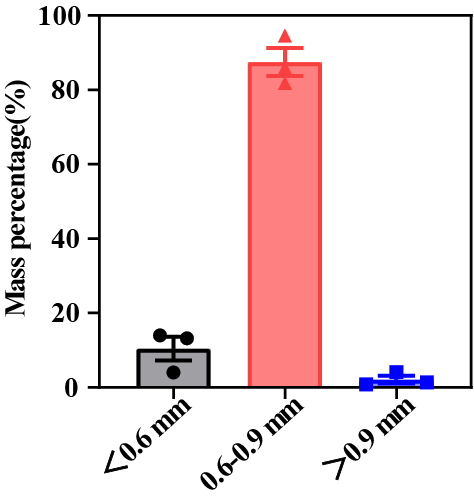
**

**Fig. S1.** Particle size distribution of the prepared pellet cores.
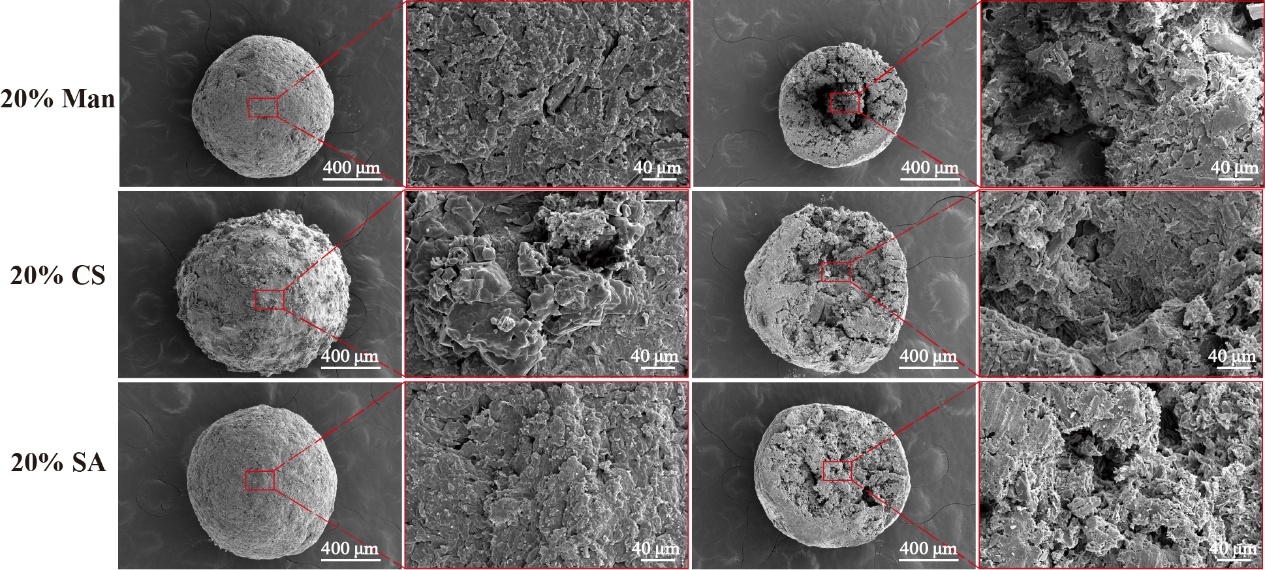


**Fig. S2.** Representative SEM images of different kinds of pellet cores.

**2. *In vitro* release analysis**

Sodium alginate (SA), a natural linear polyanionic hydrophilic polysaccharide extracted from brown algae (Phaeophyceae), has a high carboxyl group content that confers pH-responsiveness, with a decrease in solubility in acidic environments, and a maximal swelling of the structure at pH = 7.4. However, it was found in the experiment that SA@SZ-A@coated pellets (100% total weight gain) would swell and burst the coating and destroy the integrity of the coating when put into gastric juice for 1h. To investigate the reason for the expansion of the SA@SZ-A@pellet cores in gastric juice, blank SA pellet core (MCC/SA) without SZ-A, SZ-A pellet cores without SA (MCC/SZ-A), SA@SZ-A@ pellet cores (MCC/SZ-A/SA), and CS@SZ-A@pellet cores (MCC/SZ-A/CS) were prepared. They were put into artificial gastric juice to observe the swelling.

The results demonstrate that only the SA@SZ-A@pellet cores (MCC/SZ-A/SA) exhibited visible swelling within 10 min, with rapid volume expansion observed even under acidic external conditions (Fig. S2). In contrast, other pellet cores showed no significant swelling within 1 h. This phenomenon can be attributed to the water absorption and dissolution of SZ-A, which creates a localized alkaline microenvironment within the pellet cores, thereby inducing SA swelling and subsequent volumetric expansion.


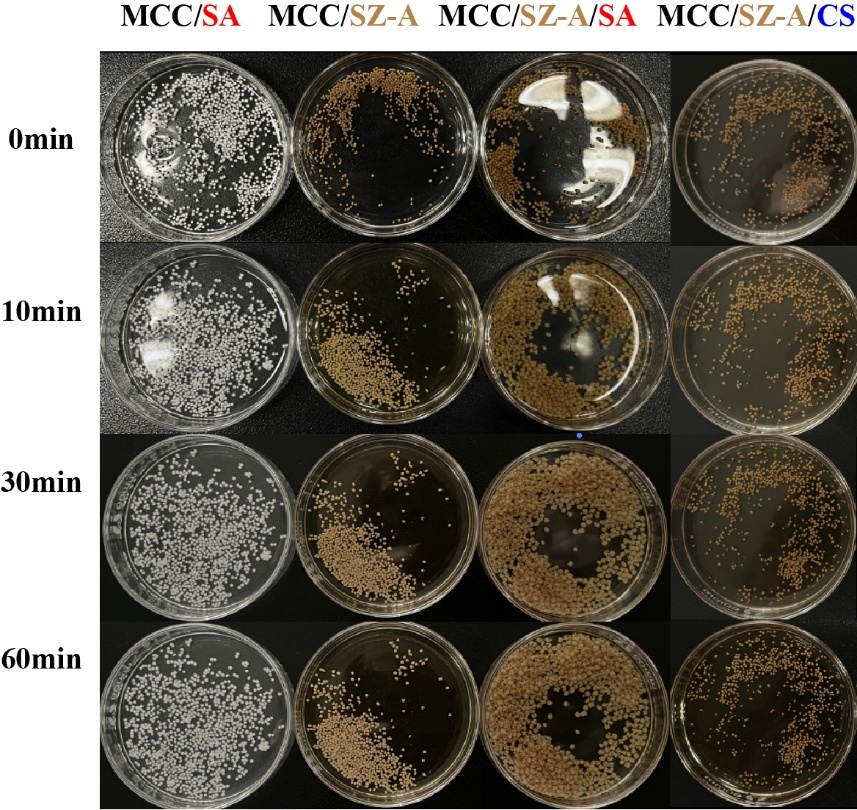


**Fig. S3.** Solubility experiment of SA@SZ-A@pellet cores in gastric fluid. In the simultaneous presence of SZ-A and SA, the pellet cores would be swollen in the presence of water due to the formation of a localized alkaline environment when SZ-A meets water, which prompted the swelling and dissolution of SA.

**3. Kinetic mechanisms**


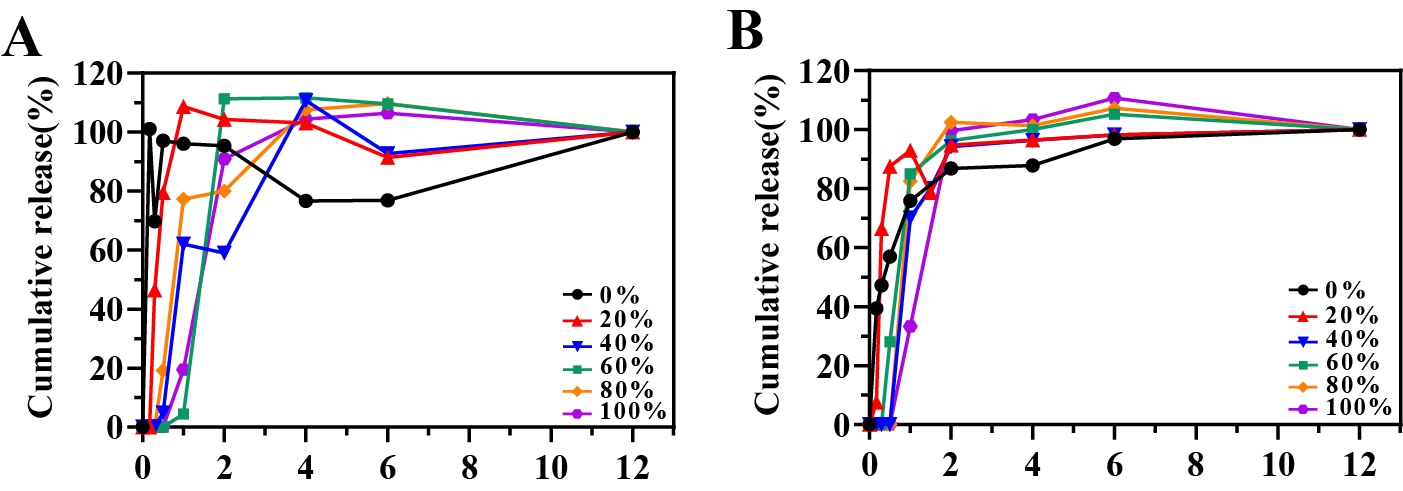


**Fig. S4.** (A) Dissolution of CS@SZ-A@pellet cores in colonic fluid. (B) Dissolution of SA@SZ-A@pellet cores in the colonic fluid.

## 4. Distribution and form of drugs


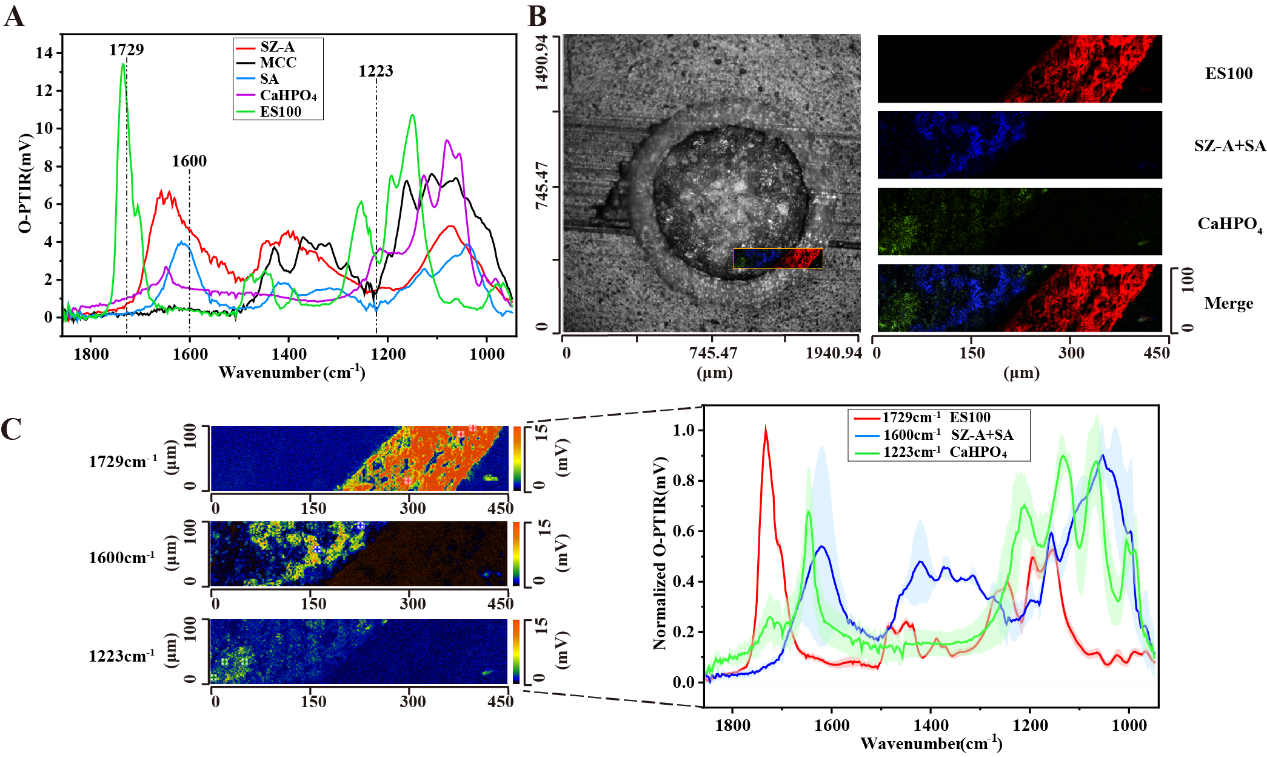


**Fig. S5.** O-PTIR was used to investigate the distribution of components in SA@SZ-A@coated pellets (100% total weight gain). (A) O-PTIR profiles of each component to determine the characteristic wavelength of a single component. (B) Optical images and overlay images of the cross-section of the pellets show that the components are evenly distributed in the pellets. (C) Chemical imaging of the pellets at specific wavebands and precise spectra at different locations (red, blue, and green dots), confirming the presence of substances through characteristic peaks.

## 5. *In vivo* targeting and retention of CS@SZ-A@coated pellets in the colon

Quantitative analysis of fluorescence signal intensity in *ex vivo* gastrointestinal tissues at the 24-hour time point was performed using Living Image software. Identical regions of interest (ROIs) were delineated for the stomach, small intestine, cecum, and colon. The relative residual fluorescence intensity percentage for each region was calculated based on ROI values, allowing comparison of fluorescence signal distribution across different gastrointestinal segments.

The results at the 24-hour time point showed that the fluorescence signals of CS@SZ-A@pellet cores (0%) were primarily concentrated in the stomach region, indicating mucoadhesive properties that delay gastrointestinal transit. In contrast, pellets with coating weight gains of 20%, 40%, and 60% exhibited fluorescence signals predominantly in the cecum and colon, demonstrating varying degrees of colon-targeting capability. Among these, the 100% group showed optimal colon-targeting efficiency with minimal fluorescence leakage in the small intestine (Fig. S2).

It should be noted that due to the substantially lower solubility of the fluorescent dye IR-820 compared to the active compound SZ-A, these quantitative fluorescence results do not fully reflect the actual in vivo distribution of SZ-A. Therefore, it cannot be concluded that 20% and 100% coating weight gains provide similar colon-targeting efficacy for the drug itself. The effect of different coating levels on SZ-A release has been confirmed through in vitro release studies, which verified that 100% coating weight gain achieves reliable colon-targeted release (Fig. 1E). The present fluorescence imaging data primarily serve to demonstrate that the coating structure itself can confer colon-targeting characteristics to the pellets, rather than enabling precise comparison of targeting efficiency between different formulations.


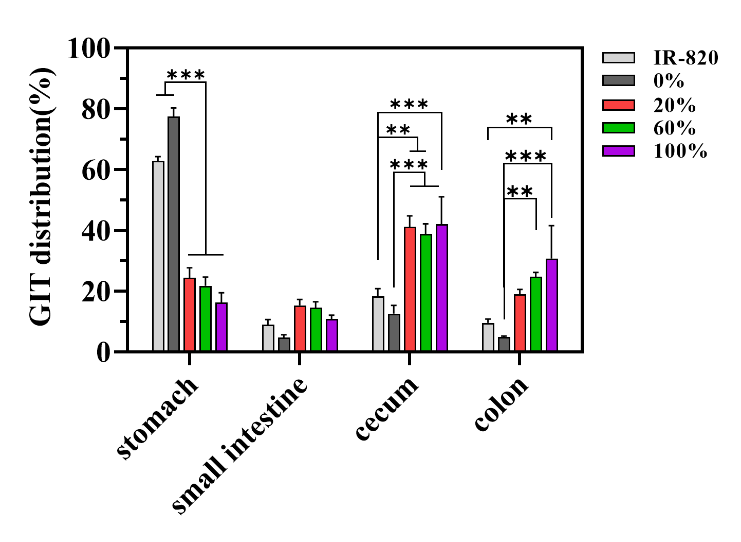


**Fig. S6.** The percentage of relative residual fluorescence intensity of each part of the gastrointestinal tract in each group of rats at 24 h.

## 6. Modulation of gut microbiome


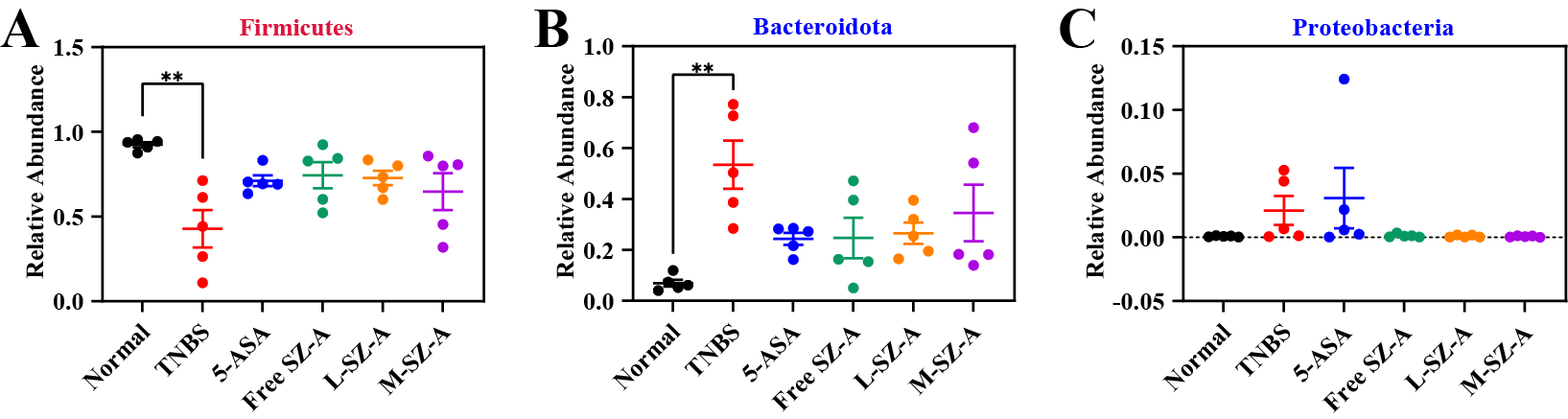


**Fig. S7.** Changes in the relative abundance of phyla-level flora. The relative abundance of (A) Firmicutes, (B) Bacteroidota, and (C) Proteobacteria. Red represented the phyla with increased relative abundance after treatment, and blue represented the phyla with decreased relative abundance after treatment. Data are expressed as mean ± S.E.M. (n=5). Significance analyzed by one-way ANOVA: *p < 0.05, **p < 0.01, ***p < 0.001.


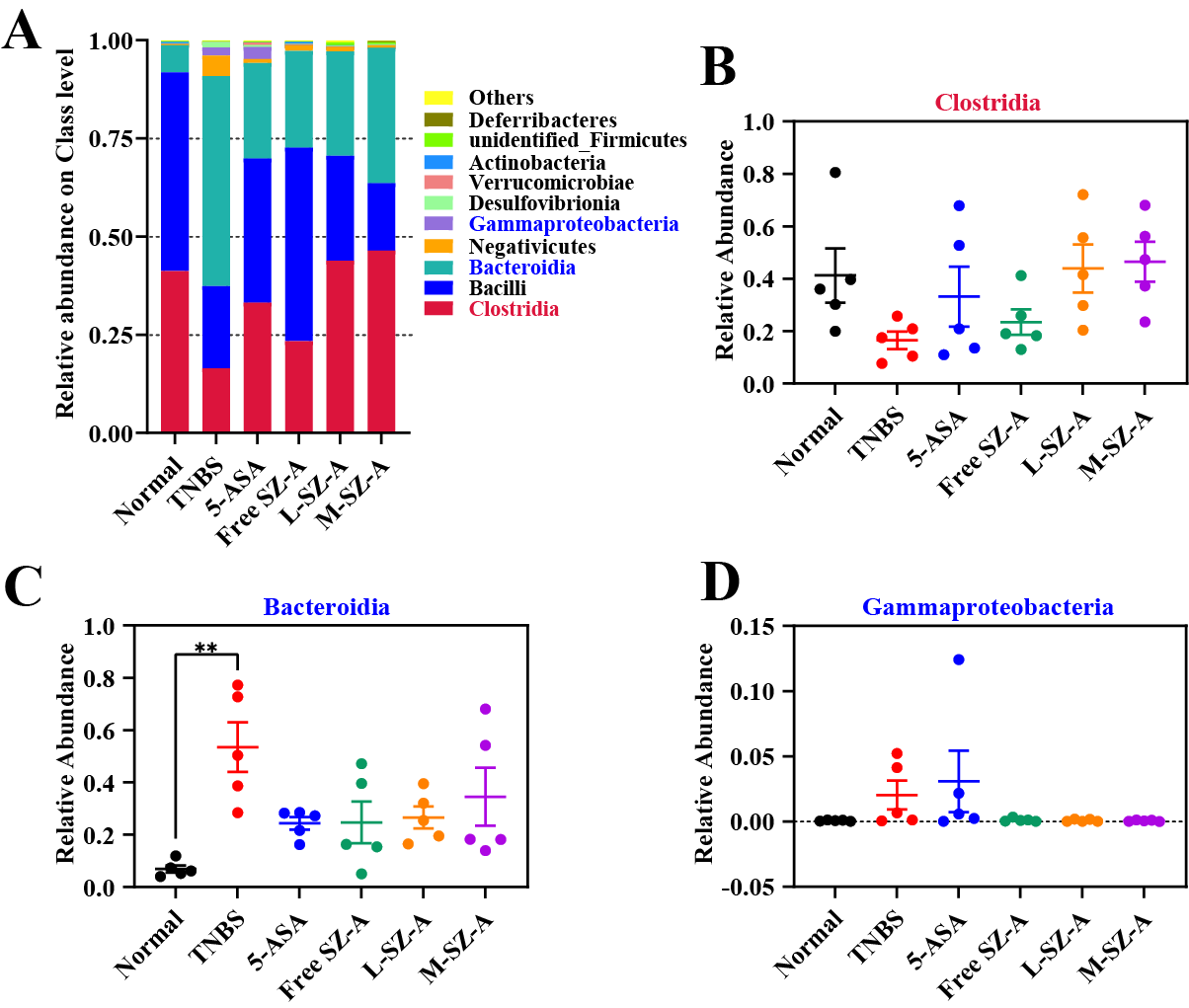


**Fig. S8.** Changes in the relative abundance of microflora at the class level. (A) Histogram of relative species abundance at the class level. The relative abundance of (B) Clostridia, (C) Bacteroidia, and (D) Gammaproteobacteria. Red indicated the bacteria whose relative abundance increased after treatment, and blue indicated the bacteria whose relative abundance decreased after treatment. Data are expressed as mean ± S.E.M. (n=5). Significance analyzed by one-way ANOVA: *p < 0.05, **p < 0.01, ***p < 0.001.


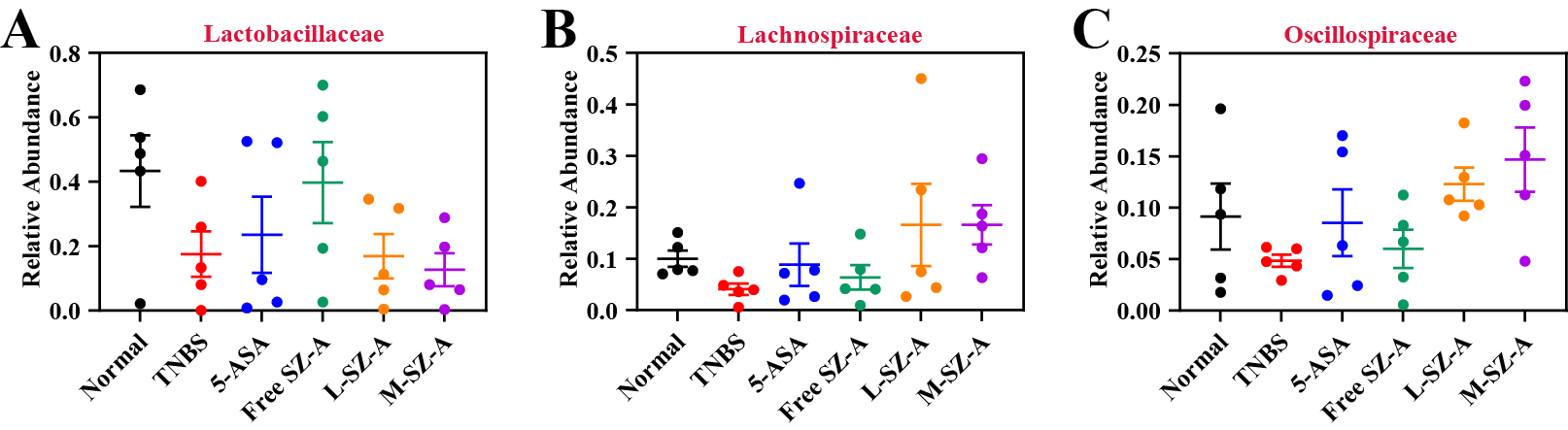


**Fig. S9.** Changes in the relative abundance of microflora at the family level. Relative abundance of (A) Lactobacillaceae, (B) Lachnospiraceae, and (C) Oscillospiraceae. Red indicated families with increased relative abundance after treatment, and blue indicated families with decreased relative abundance after treatment. Data are expressed as mean ± S.E.M. (n=5). Significance analyzed by one-way ANOVA: *p < 0.05, **p < 0.01, ***p < 0.001.

**
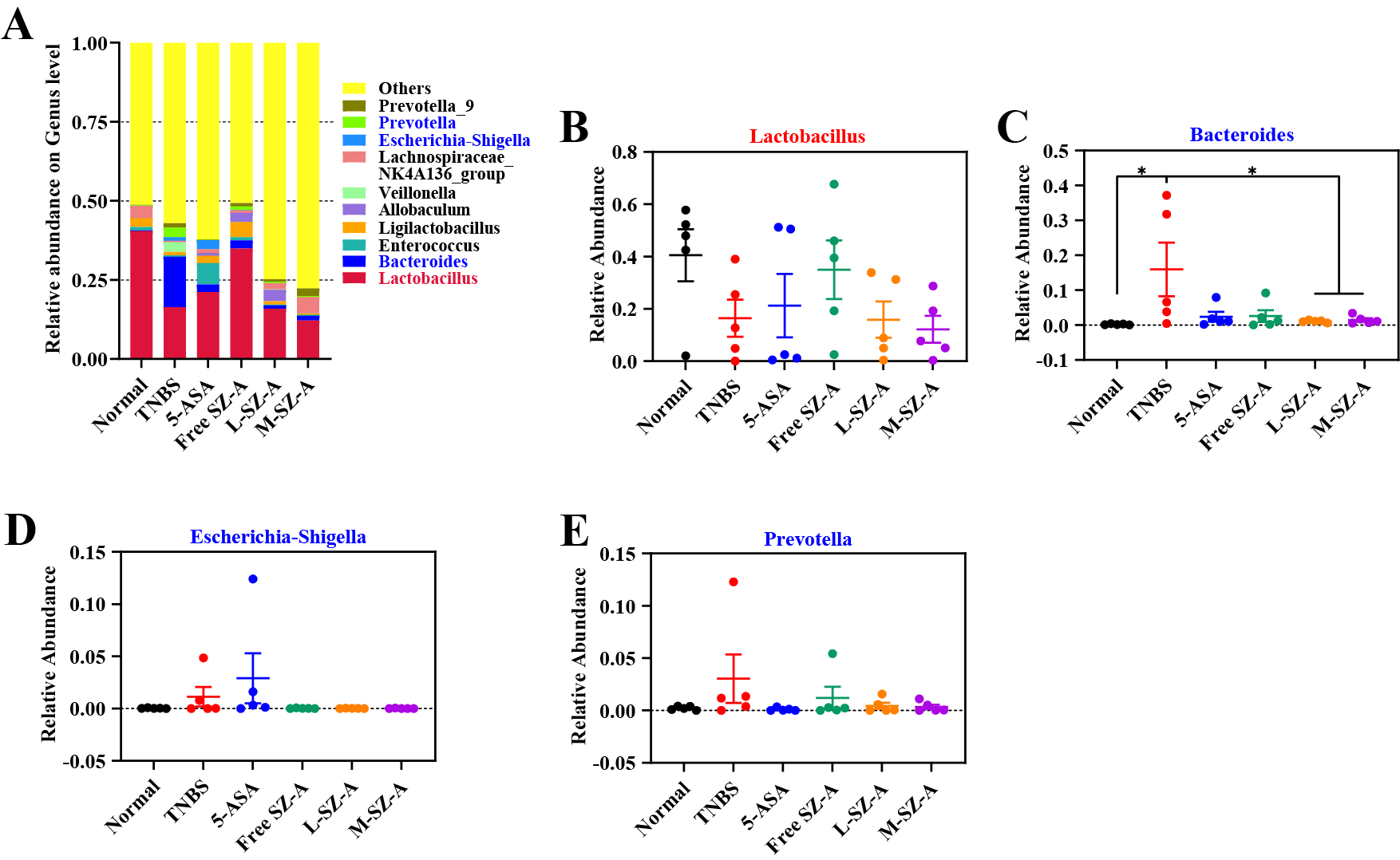
**

**Fig. S10.** Changes in the relative abundance of microflora at the genus level. (A) Histogram of relative species abundance at the genus level. Relative abundance of (B) Lactobacillus, (C) Bacteroides, (D) Escherichia-Shigella, and (E) Prevotella. Red indicated families with increased relative abundance after treatment, and blue indicated families with decreased relative abundance after treatment. Data are expressed as mean ± S.E.M. (n=5). Significance analyzed by one-way ANOVA: *p < 0.05, **p < 0.01, ***p < 0.001.

## 7. Safety assessment


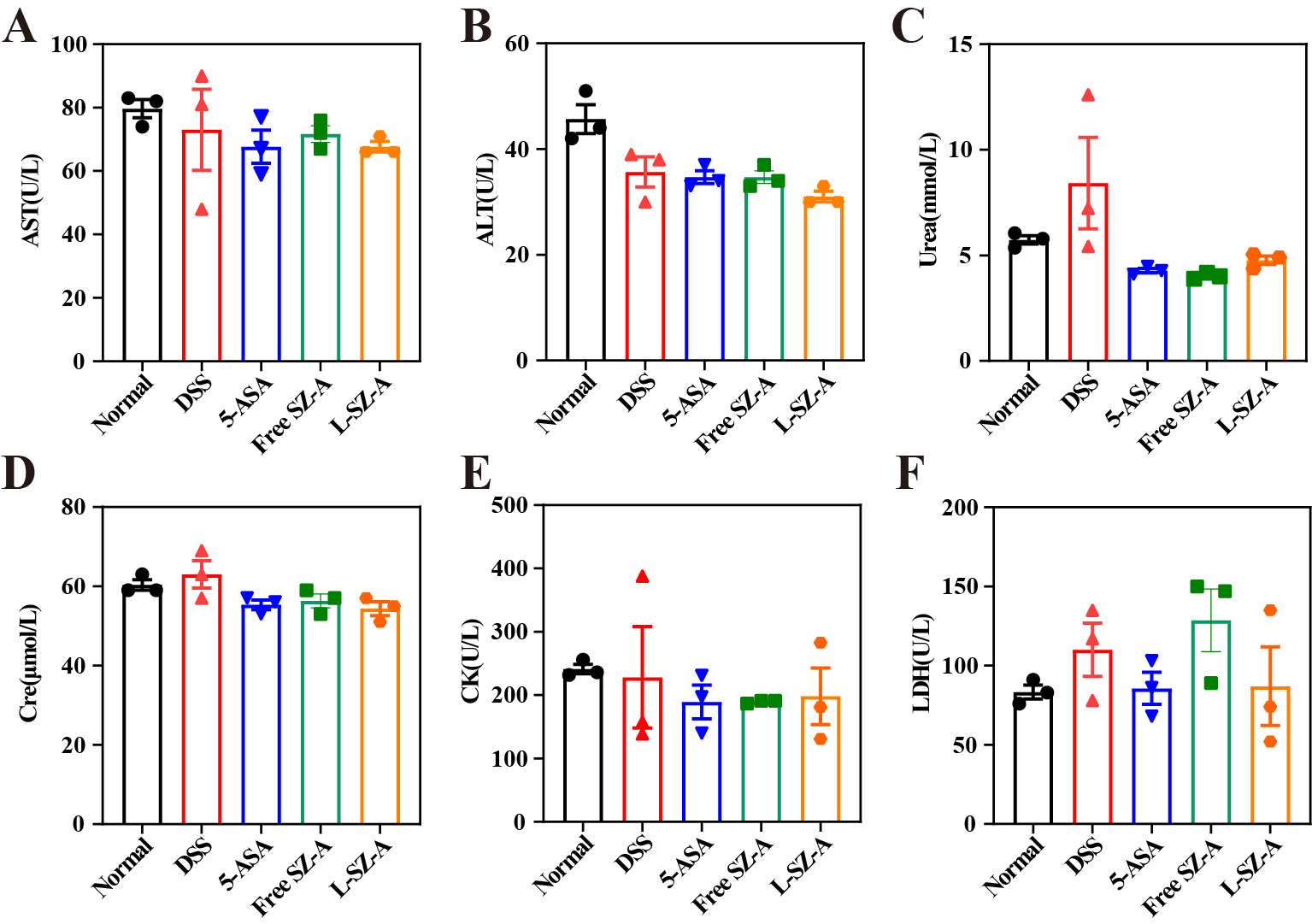


**Fig. S11.** Safety assessment of CS@SZ-A@coated pellets in the DSS-induced colitis model. Serum biochemical parameters were measured to assess key organ functions: (A-B) hepatic profile (AST, ALT), (C-D) renal function (Urea, Cre), and (E-F) cardiac and tissue integrity (CK, LDH). The data were expressed as mean ± S.E.M. (n=3), and were significant by one-way ANOVA: *p < 0.05, **p < 0.01, ***p < 0.001.

**Table S1. DAI scoring scale**

| scoring | Percentage weight loss | stool property | hematochezia |
| --- | --- | --- | --- |
| 0 | 0% | normal | no blood  (occult blood negative) |
| 1 | 1-5% | loose  (very soft but molded) | occult blood positive |
| 2 | 5-10% | semi-fluid stools  (mushy but not sticky) | visible hemorrhage |
| 3 | 10-20% | thin stools  (mucus-like, thin stools that stick to the perianal area, a little blood) | bloody stool visible to the naked eye |
| 4 | > 20% | severe watery stools with blood | obvious bloody stool |

DAI = (body mass index + stool characteristics + fecal occult blood)/3

**Table S2. Release mechanism of different kinds of pellet cores in artificial colonic fluid**

|  | Zero-level release | first-level release | Higuchi | Ritger-Peppas |
| --- | --- | --- | --- | --- |
| Man@SZ-A@pellet core | $M_{t}=0.18t+76.74$  $R^{2}=0.11462$ | $M_{t}=99.05*（1-e^{-0.70t}）$  $R^{2}=0.99813$ | $M_{t}=4.09t^{1/2}+62.34$  $R^{2}=0.2849$ | $M_{t}=96.46*t^{0.01}$  $R^{2}=0.99772$ |
| SA@SZ-A@pellet core | $M_{t}=0.49t+29.81$  $R^{2}=0.79412$ | $M_{t}=97.67*（1-e^{-0.04t}）$  $R^{2}=0.95308$ | $M_{t}=7.63t^{1/2}+10.69$  $R^{2}=0.9575$ | $M_{t}=17.02*t^{0.36}$  $R^{2}=0.98666$ |
| CS@SZ-A@pellet core | $M_{t}=0.16t+66.38$  $R^{2}=0.11159$ | $M_{t}=86.08*（1-e^{-0.54t}）$  $R^{2}=0.99893$ | $M_{t}=3.54t^{1/2}+53.80$  $R^{2}=0.2852$ | $M_{t}=82.47*t^{0.01}$  $R^{2}=0.99545$ |

**Table S3. The release mechanism of different kinds of pellet cores in artificial gastric juices**

|  | Zero-level release | first-level release | Higuchi | Ritger-Peppas |
| --- | --- | --- | --- | --- |
| Man@SZ-A@pellet core | $M_{t}=0.25t+54.61$  $R^{2}=0.14336$ | $M_{t}=73.92*（1-e^{-0.71t}）$  $R^{2}=0.99871$ | $M_{t}=4.57t^{1/2}+41.21$  $R^{2}=0.36$ | $M_{t}=71.04*t^{0.01}$  $R^{2}=0.99888$ |
| SA@SZ-A@pellet core | $M_{t}=0.47t+21.98$  $R^{2}=0.72751$ | $M_{t}=63.59*（1-e^{-0.06t}）$  $R^{2}=0.96792$ | $M_{t}=6.22t^{1/2}+8.30$  $R^{2}=0.93556$ | $M_{t}=14.80*t^{0.33}$  $R^{2}=0.98479$ |
| CS@SZ-A@pellet core | $M_{t}=0.30t+63.72$  $R^{2}=0.15377$ | $M_{t}=87.23*（1-e^{-0.56t}）$  $R^{2}=0.99924$ | $M_{t}=5.51t^{1/2}+47.65$  $R^{2}=0.37954$ | $M_{t}=81.06*t^{0.02}$  $R^{2}=0.99911$ |

**Table S4. The continuous release mechanism of SA@SZ-A@coated pellets with different total weight gain**

| TWG | Logistic | Hill | Boltzmann | Gompertz |
| --- | --- | --- | --- | --- |
| 0% | $M_{t}=138.94+\frac{-138.30}{1+{\frac{t}{2.30}}^{0.69}}$  $R^{2}=0.98645$ | $M_{t}=\frac{139.56*t^{0.68}}{{2.30}^{0.68}+t^{0.68}}$  $R^{2}=0.98641$ | $M_{t}=98.04+\frac{-280673.75}{1+e^{\frac{t+15.92}{1.98}}}$  $R^{2}=0.96703$ | $M_{t}=96.05*e^{-e^{-0.72*（t-0.73）}}$  $R^{2}=0.93984$ |
| 20% | $M_{t}=100.49+\frac{-99.63}{1+{\frac{t}{0.99}}^{3.36}}$  $R^{2}=0.99967$ | $M_{t}=\frac{100.55*t^{3.31}}{{0.98}^{3.31}+t^{3.31}}$  $R^{2}=0.99962$ | $M_{t}=99.40+\frac{-106.42}{1+e^{\frac{t-0.97}{0.33}}}$  $R^{2}=0.99838$ | $M_{t}=99.72*e^{-e^{-2.31*（t-0.83）}}$  $R^{2}=0.99978$ |
| 40% | $M_{t}=97.48+\frac{-94.68}{1+{\frac{t}{1.96}}^{7.39}}$  $R^{2}=0.99217$ | $M_{t}=\frac{97.67*t^{6.97}}{{1.94}^{6.97}+t^{6.97}}$  $R^{2}=0.99116$ | $M_{t}=96.78+\frac{-94.74166}{1+e^{\frac{t-1.96}{0.25}}}$  $R^{2}=0.98887$ | $M_{t}=97.48*e^{-e^{-2.64*（t-1.79）}}$  $R^{2}=0.99168$ |
| 60% | $M_{t}=98.58+\frac{-98.81}{1+{\frac{t}{2.26}}^{14.63}}$  $R^{2}=0.99652$ | $M_{t}=\frac{98.58*t^{14.70}}{{2.27}^{14.70}+t^{14.70}}$  $R^{2}=0.99651$ | $M_{t}=98.21+\frac{-95.68}{1+e^{\frac{t-2.48}{0.01}}}$  $R^{2}=0.99116$ | $M_{t}=98.75*e^{-e^{-4.41*（t-2.16）}}$  $R^{2}=0.99692$ |
| 80% | $M_{t}=100.27+\frac{-100.35}{1+{\frac{t}{2.66}}^{19.19}}$  $R^{2}=0.9999$ | $M_{t}=\frac{100.27*t^{19.21}}{{2.66}^{19.21}+t^{19.21}}$  $R^{2}=0.99989$ | $M_{t}=100.26+\frac{-100.44}{1+e^{\frac{t-2.67}{0.14}}}$  $R^{2}=0.99988$ | $M_{t}=100.27*e^{-e^{-5.46*（t-2.57）}}$  $R^{2}=0.9999$ |
| 100% | $M_{t}=100.00+\frac{-99.01}{1+{\frac{t}{3.98}}^{135.44}}$  $R^{2}=0.99841$ | $M_{t}=\frac{100.00*t^{178.30}}{{3.98}^{178.30}+t^{178.30}}$  $R^{2}=0.99818$ | $M_{t}=100.00+\frac{-99.01}{1+e^{\frac{t-3.98}{0.02}}}$  $R^{2}=0.99841$ | $M_{t}=100.00*e^{-e^{-9.26*（t-3.89）}}$  $R^{2}=0.99818$ |

**Table S5. The continuous release mechanism of CS@SZ-A@coated pellets with different total weight gain**

| TWG | Logistic | Hill | Boltzmann | Gompertz |
| --- | --- | --- | --- | --- |
| 0% | $M_{t}=100.36+\frac{-100.36}{1+{\frac{t}{0.06}}^{1.28}}$  $R^{2}=0.9998$ | $M_{t}=\frac{100.36*t^{1.28}}{{0.06}^{1.28}+t^{1.28}}$  $R^{2}=0.9998$ | $M_{t}=99.66+\frac{-6954.34}{1+e^{\frac{t+0.73}{0.17}}}$  $R^{2}=0.99916$ | $M_{t}=99.62*e^{-e^{-9.57*（t-0.20）}}$  $R^{2}=0.99904$ |
| 20% | $M_{t}=100.56+\frac{-96.52}{1+{\frac{t}{1.50}}^{7.34}}$  $R^{2}=0.9965$ | $M_{t}=\frac{100.81*t^{6.31}}{{1.46}^{6.31}+t^{6.31}}$  $R^{2}=0.99499$ | $M_{t}=100.34+\frac{-98.46}{1+e^{\frac{t-1.49}{0.23}}}$  $R^{2}=0.99887$ | $M_{t}=100.83*e^{-e^{-2.75*（t-1.30）}}$  $R^{2}=0.99392$ |
| 40% | $M_{t}=100.90+\frac{-99.84}{1+{\frac{t}{2.69}}^{9.72}}$  $R^{2}=0.99799$ | $M_{t}=\frac{100.93*t^{9.50}}{{2.68}^{9.50}+t^{9.50}}$  $R^{2}=0.99782$ | $M_{t}=100.74+\frac{-100.35}{1+e^{\frac{t-2.70}{0.28}}}$  $R^{2}=0.99835$ | $M_{t}=100.97*e^{-e^{-2.46*（t-2.51）}}$  $R^{2}=0.99727$ |
| 60% | $M_{t}=100.83+\frac{-101.27}{1+{\frac{t}{2.94}}^{6.70}}$  $R^{2}=0.99962$ | $M_{t}=\frac{100.78*t^{6.77}}{{2.94}^{6.77}+t^{6.77}}$  $R^{2}=0.99959$ | $M_{t}=99.82+\frac{-101.45}{1+e^{\frac{t-2.95}{0.43}}}$  $R^{2}=0.99884$ | $M_{t}=100.77*e^{-e^{-1.56*（t-2.70）}}$  $R^{2}=0.99979$ |
| 80% | $M_{t}=100.82+\frac{-100.25}{1+{\frac{t}{4.66}}^{11.10}}$  $R^{2}=0.99721$ | $M_{t}=\frac{100.89*t^{10.95}}{{4.65}^{10.95}+t^{10.95}}$  $R^{2}=0.99713$ | $M_{t}=99.98+\frac{-99.68}{1+e^{\frac{t-4.68}{0.42}}}$  $R^{2}=0.99809$ | $M_{t}=101.24*e^{-e^{-1.62*（t-4.37）}}$  $R^{2}=0.996$ |
| 100% | $M_{t}=99.49+\frac{-99.27}{1+{\frac{t}{5.68}}^{24.89}}$  $R^{2}=0.99959$ | $M_{t}=\frac{99.51*t^{24.76}}{{5.68}^{24.76}+t^{24.76}}$  $R^{2}=0.99957$ | $M_{t}=99.21+\frac{-99.06}{1+e^{\frac{t-5.69}{0.23}}}$  $R^{2}=0.99973$ | $M_{t}=99.74*e^{-e^{-3.33*（t-5.55）}}$  $R^{2}=0.99817$ |

**Table S6. The release mechanism of SA@SZ-A@coated pellets with different total weight gain in artificial colic fluid**

| TWG | Zero-level release | first-level release | Higuchi | Ritger-Peppas |
| --- | --- | --- | --- | --- |
| 0% | $M_{t}=5.75t+49.08$  $R^{2}=0.48423$ | $M_{t}=92.08*（1-e^{-2.22t}）$  $R^{2}=0.96552$ | $M_{t}=25.33t^{1/2}+31.90$  $R^{2}=0.74309$ | $M_{t}=66.91*t^{0.19}$  $R^{2}=0.95801$ |
| 20% | $M_{t}=5.12t+58.19$  $R^{2}=0.2649$ | $M_{t}=95.79*（1-e^{-2.85t}）$  $R^{2}=0.88283$ | $M_{t}=24.95t^{1/2}+39.26$  $R^{2}=0.49119$ | $M_{t}=74.12*t^{0.17}$  $R^{2}=0.73148$ |
| 40% | $M_{t}=8.37t+30.90$  $R^{2}=0.44699$ | $M_{t}=103.78*（1-e^{-0.74t}）$  $R^{2}=0.89025$ | $M_{t}=36.88t^{1/2}+5.14$  $R^{2}=0.67677$ | $M_{t}=48.32*t^{0.38}$  $R^{2}=0.71387$ |
| 60% | $M_{t}=8.21t+33.49$  $R^{2}=0.4501$ | $M_{t}=105.49*（1-e^{-0.91t}）$  $R^{2}=0.91787$ | $M_{t}=35.99t^{1/2}+9.22$  $R^{2}=0.68311$ | $M_{t}=52.65*t^{0.35}$  $R^{2}=0.73715$ |
| 80% | $M_{t}=8.80t+29.46$  $R^{2}=0.44437$ | $M_{t}=108.36*（1-e^{-0.76t}）$  $R^{2}=0.87097$ | $M_{t}=38.20t^{1/2}+3.92$  $R^{2}=0.66194$ | $M_{t}=49.22*t^{0.38}$  $R^{2}=0.69371$ |
| 100% | $M_{t}=9.65t+21.82$  $R^{2}=0.54096$ | $M_{t}=112.08*（1-e^{-0.52t}）$  $R^{2}=0.90455$ | $M_{t}=40.34t^{1/2}-4.09$  $R^{2}=0.74617$ | $M_{t}=41.93*t^{0.45}$  $R^{2}=0.74852$ |

**Table S7. The release mechanism of CS@SZ-A@coated pellets with different total weight gain in artificial colic fluid**

| TWG | Zero-level release | first-level release | Higuchi | Ritger-Peppas |
| --- | --- | --- | --- | --- |
| 0% | $M_{t}=2.25t+72.73$  $R^{2}=0.07842$ | $M_{t}=89.11*（1-e^{-4.30t}）$  $R^{2}=0.86628$ | $M_{t}=11.76t^{1/2}+63.54$  $R^{2}=0.16936$ | $M_{t}=86.39*t^{1.56}$  $R^{2}=0.85898$ |
| 20% | $M_{t}=5.57t+54.30$  $R^{2}=0.25189$ | $M_{t}=103.11*（1-e^{-2.15t}）$  $R^{2}=0.8973$ | $M_{t}=27.05t^{1/2}+34.32$  $R^{2}=0.46952$ | $M_{t}=72.01*t^{0.20}$  $R^{2}=0.6608$ |
| 40% | $M_{t}=9.04t+21.61$  $R^{2}=0.58273$ | $M_{t}=104.63*（1-e^{-0.52t}）$  $R^{2}=0.91125$ | $M_{t}=37.23t^{1/2}-1.93$  $R^{2}=0.78048$ | $M_{t}=40.21*t^{0.44}$  $R^{2}=0.78694$ |
| 60% | $M_{t}=10.05t+19.56$  $R^{2}=0.49734$ | $M_{t}=115.22*（1-e^{-0.48t}）$  $R^{2}=0.82484$ | $M_{t}=41.83t^{1/2}-7.19$  $R^{2}=0.68067$ | $M_{t}=40.28*t^{0.47}$  $R^{2}=0.67483$ |
| 80% | $M_{t}=8.78t+29.50$  $R^{2}=0.50656$ | $M_{t}=108.42*（1-e^{-0.71t}）$  $R^{2}=0.92903$ | $M_{t}=37.68t^{1/2}+4.63$  $R^{2}=0.73599$ | $M_{t}=48.97*t^{0.38}$  $R^{2}=0.7696$ |
| 100% | $M_{t}=9.82t+18.45$  $R^{2}=0.57664$ | $M_{t}=111.76*（1-e^{-0.45t}）$  $R^{2}=0.90178$ | $M_{t}=40.33t^{1/2}-6.95$  $R^{2}=0.76715$ | $M_{t}=38.29*t^{0.48}$  $R^{2}=0.75986$ |
